# Pigment-Dispersing Factor-expressing neurons convey circadian information in the honey bee brain

**DOI:** 10.1098/rsob.170224

**Published:** 2018-01-10

**Authors:** Katharina Beer, Esther Kolbe, Noa B. Kahana, Nadav Yayon, Ron Weiss, Pamela Menegazzi, Guy Bloch, Charlotte Helfrich-Förster

**Affiliations:** 1 Neurobiology and Genetics, Theodor-Boveri Institute, Biocenter, University of Würzburg, Am Hubland, 97074 Würzburg, Germany; 2 Institute of Zoology, University of Regensburg, Universitätsstraße 31, 93040 Regensburg, Germany; 3 Department of Ecology, Evolution, and Behaviour, The Alexander Silberman Institute of Life Sciences, The Hebrew University of Jerusalem, Jerusalem 91904, Israel

**Keywords:** *Apis mellifera*, circadian clock, period protein, PDF oscillation, nurse and forager bees, locomotor activity rhythm

## Abstract

Pigment-Dispersing Factor (PDF) is an important neuropeptide in the brain circadian network of *Drosophila* and other insects, but its role in bees in which the circadian clock influences complex behaviour is not well understood. We combined high-resolution neuroanatomical characterizations, quantification of PDF levels over the day and brain injections of synthetic PDF peptide to study the role of PDF in the honey bee *Apis mellifera*. We show that PDF co-localizes with the clock protein Period (PER) in a cluster of laterally located neurons and that the widespread arborizations of these PER/PDF neurons are in close vicinity to other PER-positive cells (neurons and glia). PDF-immunostaining intensity oscillates in a diurnal and circadian manner with possible influences for age or worker task on synchrony of oscillations in different brain areas. Finally, PDF injection into the area between optic lobes and the central brain at the end of the subjective day produced a consistent trend of phase-delayed circadian rhythms in locomotor activity. Altogether, these results are consistent with the hypothesis that PDF is a neuromodulator that conveys circadian information from pacemaker cells to brain centres involved in diverse functions including locomotion, time memory and sun-compass orientation.

## 1. Introduction

The remarkable time memory (Zeitgedächtnis) of foraging honey bees (*Apis mellifera*) was one of the first lines of evidence for the functional significance of the endogenous circadian clock [1,2]. It enables honey bees to synchronize flower visits with the daily floral rhythms maximizing pollen and nectar rewards [3,4]. Furthermore, in time-compensated sun-compass orientation, forager bees use the circadian clock to compensate for the movement of the sun across the sky over the course of the day [5,6]. The time-compensated sun-compass is also important for waggle dance communication, in which honey bee workers refer to the sun position in the sky when recruiting nest-mates to newly discovered floral patches or nesting cavities [7]. Plasticity in circadian rhythms is also linked to the division of labour that is important for the social organization of the honey bee colony [8].

However, the neuroanatomical organization of the bee circadian network and its connections with brain centres that control activity, sleep, memory and orientation are not well characterized. Neurons that express the neuropeptide ‘Pigment-Dispersing Factor’ (PDF) play crucial roles in the clock network of many insects and, due to their global branching pattern in the insect brain, are suitable candidates to provide time-information to these centres. Such neurons have been initially described in cockroaches, crickets [9], flies [10–12] and later in a variety of other insects including bees [13–16]. In cockroaches, crickets and flies, the PDF neurons have dense arborizations in a small neuropil at the base of the medulla that is called accessory medulla and is regarded as the circadian pacemaker centre in these insects [17,18]. In flies and honey bees, the PDF neurons express the clock protein Period (PER) and can therefore be regarded as bona fide clock neurons [19,20].

PDF neurons have been shown to be essential for circadian rhythms in several insects, including species in which co-expression of PER and PDF has not been reported so far [17,18,21–26]. In fruit flies, the intensity of PDF-immunostaining as well as the arborization complexity of the PDF terminals in the central brain was shown to oscillate during the day, which was interpreted as circadian release of PDF [27–29]. Besides being an output factor of the fly circadian clock, PDF also functions as a key communication factor within the circadian network [30–32]. Consistent with this premise, most clock neurons express PDF receptors allowing them to respond to the PDF signal [33]. In cockroaches and crickets, injections of PDF into the optic lobes phase-shifted rhythms in locomotor activity and in visual sensitivity, which is consistent with similar circadian functions in these insects [34–37]. In honey bees *Pdf* mRNA levels oscillate, but cycling of the peptide has not yet been reported [15].

This study tests the hypothesis that PDF signalling is involved in communicating circadian information in the honey bee brain. To meet this goal, we performed a three-dimensional (3D) characterization of PER and PDF neurons in the honey bee brain and studied PDF-staining intensity in selected fibres as a function of time of day. Given that circadian rhythms in honey bee relates to worker task (division of labour [8]), we determined cycling for both behaviourally rhythmic foragers and behaviourally arrhythmic nurses. Finally, we injected synthetic honey bee PDF peptide into the honey bee brain to test whether it affects circadian rhythms in locomotor activity.

## 2. Material and methods

### 2.1. Honey bee colonies

Honey bee colonies used for our studies were kept according to standard beekeeping practices at the Hebrew University of Jerusalem (Israel), the University of Regensburg (Germany) and the University of Würzburg (Germany). Each stock was composed of a mixture of subspecies typical to their region. If not stated otherwise, the queens of the colonies we studied mated naturally (with multiple drones). To obtain bees of known age we removed honeycombs with newly emerging bees and late stage pupae (identified by their relatively dark body pigmentation and purple to dark eyes [38,39]). The adult bees were removed and the brood comb was transferred in a lightproof cage into an incubator (33° ± 1°C, 55 ± 5% relative humidity) for 24 h. After 24 h, we collected newly emerging bees (0–24 h of age), marked them with a dot of coloured paint (Testor's Enamel) on the dorsal part of the thorax between the wings and introduced them back into their mother colony.

### 2.2. Detailed characterization of protein Period and Pigment-Dispersing Factor immunostaining

#### 2.2.1. Pigment-Dispersing Factor immunostaining and 3D reconstructions

For the 3D description of PDF immunostaining we collected forager bees of unknown age from one colony at the Department of Zoology in Regensburg. Forager bees returning to the hive with pollen loads were collected at the entrances at the late afternoon during spring (approx. 12 h day length), and were immediately dissected and processed for fluorescent immunocytochemistry. Whole brains were fixed overnight in Zamboni's fixative (4% paraformaldehyde, 7.5% saturated picric acid solution in 0.1 M PBS, pH 7.4), rinsed (3 × 10 min) in phosphate-buffered saline (PBS, 137 mM NaCl, 2.7 mM KCl, 10 mM Na_2_HPO_4,_ 1.8 mM KH_2_PO_4_, pH 7.4) and rinsed (3 × 10 min) in PBS containing 0.5% Triton (PBST 0.5%). They were subsequently blocked in 5% normal goat serum in PBS overnight at 4°C. Polyclonal anti-β-PDH raised in rabbits against the crab β-pigment-dispersing hormone (PDH [40]; kindly provided by Heinrich Dircksen) was applied at 1 : 3000 (in PBST) for 5 days in order to guarantee that the antibody penetrated throughout the brain. This crab antibody reliably stains the PDF-positive neurons of insects, including honey bees [9,19,20]. After 4 days incubation with the secondary fluorescent antibody (Alexa Fluor 555, goat anti-rabbit, 1 : 200) the brains were incubated over night with Lucifer Yellow (1 : 12 800) to stain the neuropils. The brains were washed with PBS (5 × 10 min), dehydrated in ethanol (30, 50, 70, 90, 95, 3 × 100%; each step 20 min), cleared in methyl benzoate (50 : 50 ethanol : methyl benzoate for 20 min, 100% methyl benzoate overnight) and embedded in Permount (Fisher Scientific, Schwerte, Germany) between two coverslips separated from each other with spacers to prevent squeezing of the brains.

Confocal images were obtained using a Leica TCS SPE confocal microscope with a 10× air objective (numerical aperture: 0.3). The whole-mount brains were scanned at a resolution of 1024 × 1024 in the *xy* direction and a step size of 2.39 µm in the axial direction. Because of the thickness of the brains, each brain was scanned in anterior and posterior image stacks, which were later aligned in *z*-direction in the Amira software (v. 4.1.1) by using the landmark tool.

One brain showing the best PDF staining (out of five) was reconstructed with Amira. Neuropils were reconstructed in the segmentation editor and, after subsequent resampling, a smoothed surface 3D model was generated. PDF fibres were reconstructed using the ‘neuron tracer plugin’. Here we added a high number of tracing points along the fibres and smoothed reconstructed structures in order to avoid calculation errors in dense fibre tracts. The varicosities of the PDF fibres are generally not preserved in the neural network reconstructions. To demonstrate the varicosities, we show original confocal images of certain brain areas in addition to the reconstructions.

#### 2.2.2. Protein Period and Pigment-Dispersing Factor double staining

The PER and PDF double staining experiments were performed in Würzburg. We generated 14 preparations of whole-mount brains and vibratome sections based on honey bees from two colonies kept at the Department of Animal Ecology and Tropical Biology. Pollen forager bees were collected at the hive entrances in the late afternoon during spring and summer—LD was approximately 11 : 13 h in the whole-mount experiments (sunset = 18.00), and approximately 16.5 : 7.5 h for the vibratome experiments (sunset = 21:30)—and placed inside an incubator until sampling (20 ± 0.5°C, 60 ± 10% humidity, approximately 200 lux during light phase; light was switched off at natural sunset). They were sacrificed during their first night in the incubator 2 h before the time of sunrise, which mimics the time of high PER [14,20], and immediately processed for immunocytochemistry. PER was detected by an antibody raised against the entire *am*PER molecule, characterized in [20] and provided by courtesy of Eva Winnebeck. Since both antibodies, anti-*am*PER and anti-βPDH, were raised in rabbit [20] we performed two separate staining procedures with an additional fixation in Zamboni's fixative in between (for details, see [20]). This additional fixation of the brain samples in Zamboni's fixative is assumed to denature remaining free binding sites of the anti-PER antibody which successfully prevented that the second secondary antibody recognized PER and allowed us to unequivocally distinguish between PER and PDF staining. The incubation in anti-PER solution was 7 days for whole-mounts and 2–4 days for brain sections, both at 4°C (anti-PER was diluted 1 : 1000 in PBST for whole-mounts and 60 µm vibratome sections; in 150 µm vibratome sections the antibody was preabsorbed on *per^0^ Drosophila melanogaster* mutants to reduce non-specific staining, and was diluted 1 : 100). The incubation in anti-β-PDH was 7 days for whole-mounts and 4 days for sections (dilution 1 : 3000). One representative detail scan of a whole-mount brain showing PER- and PDF staining in the lateral brain was used to reconstruct the projections of the PDF neurites in relation to the PER-positive nuclei with Amira software (version 6.1.1) using the ‘filament tracer’ tool.

#### 2.2.3. Additional horseradish peroxidase and DAPI staining

Some of the PER and PDF immunostained vibratome sections were additionally labelled with a fluorochrome-coupled anti-HRP (horseradish peroxidase) antibody that immunostains the neuropils and the somata of neurons. The HRP antibody recognizes a carbohydrate residue of the neuron-specific cell surface protein Nervana [41]. Therefore, HRP labels the surface of all neurons (cell bodies and neurites) but not glial cells, and leaves the nuclei unlabelled [42]. For the HRP staining, we incubated the tissue with Cy3-AffiniPure Goat Anti-HRP (catalog no. 123-165-021, Jackson ImmunoResearch, West Grove, PA, USA) diluted at 1 : 300 in PBST 0.5% (with 5% NGS and 0.02% NaN_3_) for 48 h at room temperature and washed five times in PBST (0.5% Triton). In the fifth washing step we added DAPI (4-6-diamidino-2-phenylindole dihydrochloride hydrate; Sigma D9542; Eugene, Oregon, USA; 1 mg ml^−1^ washing solution) to allow for nuclear counterstaining, and washed three times in PBS (see [20]). Afterwards the sections were mounted in Vectashield.

#### 2.2.4. Nomenclature

We followed the naming conventions for neuropils suggested by Ito *et al*. [43] wherever possible. The naming of the clock neurons is adapted to *Drosophila melanogaster* and our recent definitions for *A. mellifera* [20]. For simplicity, we will refer to the neurons that are stained with the PER antibody and/or the PDH (PDF) antibody ‘PER-positive’ and ‘PDF-positive’ neurons, or simply ‘PER and PDF neurons’, respectively. Similarly, we will refer to fibres arising from the PDF neurons as ‘PDF fibres’.

### 2.3. Diurnal and circadian variation in Pigment-Dispersing Factor staining

The first two experimental trials were performed with bees from the Hebrew University of Jerusalem in October (11.6 h day length). The queens of the colonies used for these experiments were inseminated with the semen of a single drone (different for each colony) in order to reduce genetic variation among daughter workers (honey bee queens naturally mate with 10–20 males) in the colony. Foragers and nurses were collected either every six hours (ZT4, ZT10, ZT16 and ZT22, whereby ZT0 is defined as sunrise) or every four hours (ZT2, ZT6, ZT10, ZT14, ZT18 and ZT22) directly from a hive, in which the foragers could fly outside and were entrained by the natural light–dark cycle. Forager bees were identified by their conspicuous pollen loads and were paint marked during the day to enable identification at times when not foraging (e.g. during the night). Nurses were worker bees observed with their heads inside a brood containing honeycomb cell. Brains were quickly dissected, fixed and shipped in the fixative solution to Regensburg for immunofluorescent treatment (see below).

Experiments three and four were entirely performed in Regensburg during spring (day length 12.5 h) and included only foragers (identified as above). Since there was no possibility to access the hive at night, the foragers were collected directly at the entrance of a single hive during the evening, just before sunset (approx. 18:00). The collected bees were immediately transferred to an incubator with a 12 : 12 LD regime, in which the light was switched off at natural sunset (28 ± 0.5°C, 80 ± 2% humidity). In experiment three, the bees were entrained for 3 days and then dissected at six different time points (ZT2, ZT6, ZT10, ZT14, ZT18 and ZT22), while in the fourth timeline experiment, bees were kept in constant darkness (DD) for 3 days and then dissected on the fourth day every 4 h.

#### 2.3.1. Anti-β-Pigment-Dispersing Factor staining for samples collected around-the-day

For quantitatively analysing the β-PDH staining intensity, all brains within one experiment were processed together and treated exactly in the same way (keeping times of fixation, rinsing and antibody incubation constant). The brains were fixed in Zamboni's fixative for 4 days, rinsed and embedded in gelatine–albumin: 12 g gelatine (Sigma, 300 bloom), 75 g ovalbumin (Sigma, A 5253, albumin chicken egg grade III) in 250 ml distilled water. The gelatine–albumin blocks containing the brains were fixed again in 4% PFA at 4°C overnight and then stored in PBS. The blocks were cut with a vibratome (Leica, VT1000 S) in 100 µm-thick frontal sections. Fluorescent anti-β-PDH staining was then performed on the floating vibratome sections as stated in ‘PDF immunostaining’ but with shorter antibody incubation times (anti-β-PDH 1 : 3000 for 48 h, ALEXA Fluor 488 goat anti-rabbit 1 : 200 overnight).

#### 2.3.2. Analysis of Pigment-Dispersing Factor staining intensity

PDF staining intensity of the first two experiments (four and six time points under naturally synchronized conditions; bees from Jerusalem) was quantified as described by Park *et al*. [27]. Briefly, the area of interest was chosen for each brain. The average intensity in grey values of 10 points (pixels) in the background close to stained structures was subtracted from the average intensity of the 10 brightest points in PDF stained structures. In experiments three and four, the 10 brightest points were close to saturation (255 pixels) at the maxima of PDF cycling. Therefore, we quantified staining intensity by using the protocol described by Hermann-Luibl *et al*. [29]. Briefly, 10 confocal stacks containing the relevant PDF-structure were merged and background was set to zero by adjusting ‘intensity’ of the image in Corel Photopaint (version X6, 64 bit). All labelling outside the relevant structure was manually removed and the pixel intensity of the entire image determined in ImageJ. If not stated otherwise, staining intensity was determined in the relevant structure of both brain hemispheres and an average staining intensity calculated for each brain.

#### 2.3.3. Statistics

First, we tested whether the data were normally distributed by the Kolmogorov–Smirnov test. If this was the case, we applied a one-way analysis of variance (ANOVA) to test for significant influences of time-of-day on PDF staining intensity. In cases in which data were not normally distributed, we used a non-parametric Kruskal–Wallis test. Furthermore, in all cases in which ANOVA or the Kruskal–Wallis test did not reveal significant time-of-day influences, we applied JTK_CYCLE (version 3.4), a non-parametric algorithm for detecting rhythms in transcripts [44].

### 2.4. The influence of Pigment-Dispersing Factor injections into the brain on circadian rhythms in locomotor activity

These experiments were performed at the Hebrew University of Jerusalem. We used foragers (identified as described above), or forager-age (21–22 days old) bees, which typically have strong circadian rhythms (reviewed in [8,45]). We first performed a set of preliminary experiments in which we confirmed that cold anaesthesia and the injection procedure in our protocol do not affect bee survival and circadian rhythmicity (data not shown). For the main experiments, the bees were collected from two colonies: one with a naturally mated queen, and the other with a queen instrumentally inseminated with semen of a single drone (to reduce genetic variation within the colony).

#### 2.4.1. Monitoring of locomotor activity and determining the effects of Pigment-Dispersing Factor on circadian rhythmicity

We collected 11-day-old bees into wooden cages and synchronized the bees for 4 days in a LD 12 : 12 regime. During the dark phase we transferred them in a light sealed box into an environmental chamber (28°C, 60% relative humidity) with constant dim red light (greater than 640 nm, peak at around 680). Each bee was placed individually in a monitoring cage made of a modified Petri dish (9 cm diameter, 1.5 cm height), provisioned with 50% sugar water. We monitored locomotor activity with the ClockLab data acquisition system (Actimetrics CO., USA) with four light-sensitive black and white Panasonic WV-BP334, 0.08 lux CCD cameras (each camera recorded activity from 30 cages), and a high-quality monochrome image acquisition board (IMAQ 1409, National Instruments Co., USA) [39,46]. Data were collected continuously at a frequency of 1 Hz. On the fifth day after transfer into the locomotor activity monitoring system, we injected the bees either with saline or with PDF dissolved in saline (see below). An additional control group included bees that were similarly handled and anaesthetized but were not injected. All manipulations were done 8–11 h after the onset of the bees' locomotor activity. This corresponds to circadian time (CT) 8–11 (= CT8–11) and is around the end of their subjective day, a time at which we expect prominent phase delays according to studies performed in cockroaches [34,35]. The injected bees were returned to the monitoring system and their activity was recorded for additional four days in constant dim red light. To calculate putative phase shifts induced by the treatment, we used the ClockLab software to add linear regression lines to the daily time of onset of activity during the four days before, and the four days after the day of injection. The difference between the interpolations of the two regressions lines on the day following the day of injection was used for estimating the phase shift. We also compared the days before and after injection in terms of: (1) the free-running period and (2) the strength of circadian rhythms (Power). These indices were determined with the ClockLab software before and after the injections. Then, changes in period (ΔFRP) and power (ΔPower) were calculated by subtracting the values before the injections from those afterwards. The effect of the treatment was analysed using the non-parametric Mann–Whitney test (SPSS Inc.).

#### 2.4.2. Synthetic Pigment-Dispersing Factor peptide

The amidated *A. mellifera* Pigment-Dispersing Factor (*Am*PDF) was synthesized by BioSight Ltd. Peptide Technologies (Carmiel, Israel; product no. 07-01-0013-1) based on the PDF orthologue sequence predicted in the honey bee genome (NSELINSLLGLPKNMNNA—Amide [47]). The peptide was dissolved in ultra-pure water to reach a concentration of 2 mM PDF, divided to 100 µl aliquots and frozen in −80°C until use. We performed mass spectrometry and HPLC tests to determine the purity and estimate the amount of PDF peptide in the solution before using the peptide. The amount of injected PDF was corrected based on these estimations. The peptide solution was mixed with honey bee saline to reach a final concentration of 0.001 mM PDF, 0.01 mM PDF and 0.1 mM PDF. Control bees were injected with saline alone that was prepared by a standard protocol [48] and kept at −4°C before use.

#### 2.4.3. Pigment-Dispersing Factor and saline injections

We used two different injection protocols. The first protocol was based on similar injection studies with cockroaches [34] and crickets [36], and requires the removal of parts of the head capsule cuticle. In the second, newly developed protocol, we punctured a small opening in the head cuticle without removing it. In both cases, we injected only into the right optic lobe, whereas the left lobe was left intact. Furthermore, in both cases the bees were anesthetized by chilling them down to a temperature of approximately 1°C. The whole procedure was performed under a dissecting microscope equipped with red light illumination (optic fibre system with red light filters; greater than 640 nm, peak at around 680). Care was taken not to expose the bees to white light.

The injections themselves were performed using an electric nanoliter injector (Nanoject II, Drummond, cat. no. 3-000-205) loaded with a glass electrode (Drummond 3.5″, cat. no. 3-00-203-G/X) pulled with a micropipette puller P-97 (Sutter Instruments Co.). The electrode was first filled with heavy mineral oil (Sigma) that is not compressible, enabling a more accurate volume injection and then loaded with approximately 50 µl of injection solution. The micro-injector was directed with a mechanical micromanipulator (Right 3-000-024-R) to the desired position.

#### 2.4.4. Injections after cutting a window into the head capsule

The bee was fixed with a strip of commercial Play-Doh that was placed over the head capsule on a custom-made ice-chilled injection platform. We cut a small window in the head capsule using a delicate scalpel and fine tweezers. We first cut two incisions through the cuticle, the first incision starting between the antennae base and ending between the ocelli and the tip of the compound eye, and the second along the frontal side of the compound eye until merging with the first incision at the dorsal tip of the head. Then, the cuticle piece, which was still connected to the bee head at its base, was flapped down and held in its position with a stapling pin. The brain and surrounding tissues was now visible. The electrode was directed to the middle of the vertical axis passing through the lobula, which was easy to locate. Once in position, the electrode was inserted, penetrating the brain tissue to a 20 µm depth from its external surface (as measured by the turning of the micromanipulators’ small screw). Of note, 2.3 nl of the solution was injected into the tissue and the electrode was slowly retracted from the brain. Following injection, we reattached the piece of cuticle by placing hot commercial bee wax (obtained from Yad Mordechai Apiary, Israel) over the incision. The entire procedure, from anaesthesia to the sealing of the cuticle, lasted between 7 and 15 min.

#### 2.4.5. Stereotactic injections through the head capsule cuticle without cutting a window

For this protocol, we designed and built a new custom-made aluminium fixation mould, which enabled uniform fixation of the bee in such a way that the anterior surface of the head was perpendicular to the nano-injector (electronic supplementary material, figure S1a–c). The fixation mould was designed with a wide base to allow efficient and uniform chilling (approx. 1°C) of the bee without wetting (electronic supplementary material, figure S1a,c). The bee was fixed on the mould by inserting its neck trough a narrow cleft (electronic supplementary material, figure S1c) and harnessing her with soft Play-Doh strips on the head and back. Injection site was determined with the help of a gridded stereomicroscope lens (using ×40 magnification). The vertical scale of the grid was oriented in such a way that it reached from medial ocellus until the area in between the origin of the two antenna (electronic supplementary material, figure S1d). The ocellus was placed exactly at the vertical scale mark 25, and the injection was done at scale mark 25 on the left arm of the grid (electronic supplementary material, figure S1d). The cuticle was pierced with an entomological metal pin, and then the tip of the electrode was inserted 1.2 mm from the cuticle surface into the bee brain tissue. The electrode was then retracted by 0.2 mm and two consecutive injections of 2.3 nl were made (total = 4.6 nl). Next, the electrode was fully retracted out of the bee's head and the bee was immediately placed in its cage for recuperation. The entire stereotactic injection procedure took only 2.5 min, which is significantly shorter than the first procedure.

#### 2.4.6. Verifying the site of injection

For the first protocol, in which injection site was visually determined based on brain morphology, we verified the anatomical location by using the same injection protocol but with a fluorescent dye (DiI, Molecular probes, D-3911 diluted 1 : 1000 in ethanol). Following injection, we fixed the brains and performed fluorescent PDF immunocytochemistry as described above.

For the second protocol, we estimated the anatomical site of injection retroactively. We mixed the solutions with 2% blue dye (Chicago Sky Blue powder 6B, SN. C8679; Sigma LTD) to reveal the injection site. This dye binds covalently to the cell membrane for up to 2 weeks. It is commonly used in neurobiology and is not known to have notable effects on cell function [49].

At the end of the experiment (five days post injection), we sacrificed the bee and opened the head capsule to expose the brain. We photographed the brain of each bee with a digital camera (Nikon) under ×50 magnification Then, each injection mark on the photograph was digitally measured and given *x* and *y* coordinates. The distance between the two saddles (i.e. between the optic lobe and central brain) was measured and used as a normalization factor allowing precise site localization for bees differing in brain size. All the normalized coordinates were combined and displayed on a reference figure of a brain stained with anti-PDF antibody, using a specific algorithm (Matlab). Then, the presented data were combined with an actual anatomical image that was dyed against PDF (Adobe Illustrator CS4). This imaging enabled us to crudely estimate the effect of the site of injection relative to PDF-positive neurons on circadian rhythms in locomotor activity.

## 3. Results

### 3.1. The Pigment-Dispersing Factor neurons comprise a subgroup of the protein Period-positive neurons that arborize throughout the brain

**Figure 1.**
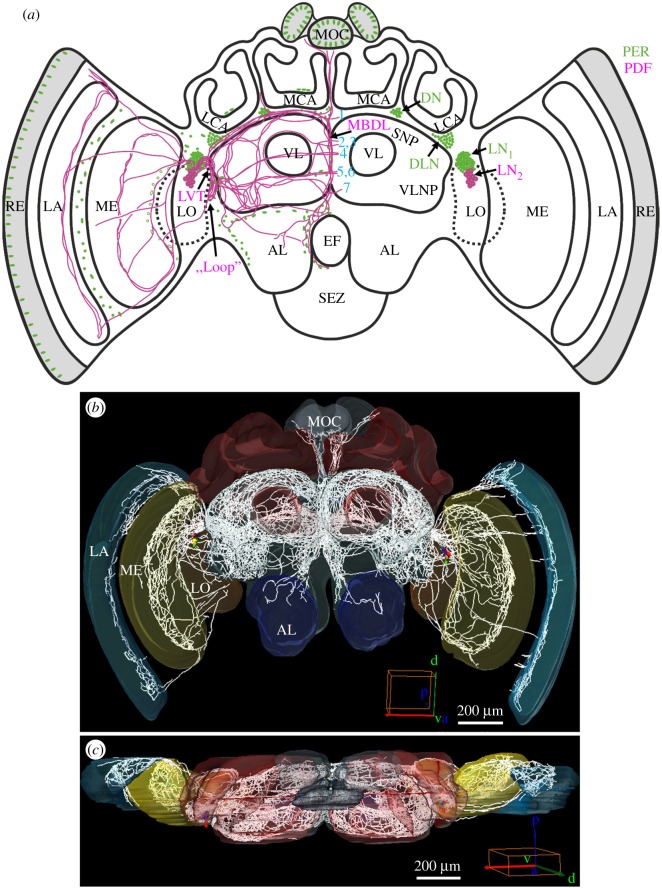
PER- and PDF-positive cells in the brain of the honey bee *A. mellifera*. (*a*) Schematic presentation of the PER (green) and PER/PDF-positive cells (magenta) in the brain. The main fibre tracts of the PER/PDF-positive neurons (magenta) are additionally depicted in the left brain hemisphere together with the nuclei of PER-positive glia cells and photoreceptor cells (green). Abbreviations: 1–7, commissures 1–7; AL, antennal lobe; DLN, dorsolateral neurons; DN, dorsal neurons; EF, oesophageal foramen; LA, lamina; LCA, lateral calyx; LN_1_, lateral neurons 1; LN_2_, lateral neurons 2; LO, lobula; LVT, lobula valley tract; MBDL, median bundle; MCA, medial calyx; ME, medulla; MOC, medial ocellus; RE, retina; SEZ, suboesophageal zone; SNP, superior neuropils; VL, vertical lobe; VLNP, ventrolateral neuropils. (*b,c*) Reconstruction of all PDF fibres which emanate from the PER-/PDF-positive LN_2_ in a frontal (anterior) view (*b*) and from a dorsal view (*c*). Cell bodies are coloured, fibres are depicted in white. The PDF fibres invade all optic ganglia (LA, lamina; ME, medulla; LO, lobula), the ocelli (MOC, medial ocellus) and most parts of the bee's protocerebrum, as well as (sparsely) the ALs of the deutocerebrum. In addition, the tritocerebrum shows PDF-positive fibres around the oesophageal foramen that are hard to see in these reconstructions. Mushroom bodies and the central complex are free of PDF fibres.

The clock protein PER is expressed in four major neuronal clusters in the lateral and dorsal brain of the honey bee (figure 1*a*) [20]. PDF co-localizes with PER in the lateral neurons 2 (LN_2_) cluster that in foragers consists on average of 15.2 (±0.4) neurons (*n* = 16, all brains sampled at one time point with high PDF levels) with rather large somata (figure 1*a*). The PDF fibres emanating from the LN_2_ cluster invade most parts of the brain, including the optic lobes, the ocellar tract and the antennal lobes (ALs), and with a particularly dense branching pattern in the protocerebrum (figure 1*b*,*c*; see also [14] and [15]). Owing to the massive overlap of the PDF fibres, it was impossible to unambiguously assign projections to individual neurons. Nevertheless, using detailed 3D reconstruction we were able to unravel neuroanatomical details that have not been described before, neither in sections [14] nor in whole-mount labelling [15]. Below, we describe novel features in the projection pattern of the entire population of PDF neurons with emphasis on fibres passing close to PER-positive cells, adjacent to the mushroom bodies or to the central/lateral complex, and in brain regions in which we later recorded PDF-immunostaining intensity. A more detailed description of the PDF projections can be found in [50] and a movie demonstrates the PDF fibres in 3D (Movie PDF-network (2).avi; https://doi.org/10.5281/zenodo.999523).

### 3.2. Projection pattern of the Pigment-Dispersing Factor neurons

#### 3.2.1. Fibres of the Pigment-Dispersing Factor neurons form a high-density Pigment-Dispersing Factor-network in front of the lobula

The somata of the bee PDF neurons were located proximally of the medulla and are heterogeneous in size. One PDF neuron with rather big soma was located most anteriorly (shown in red in all 3D reconstructions). The remaining somata could hardly be classified into size categories; they varied in width from 7–21 µm and in length from 10–29 µm, as already reported by Bloch *et al*. [14] and Fuchikawa *et al*. [20].

**Figure 2.**
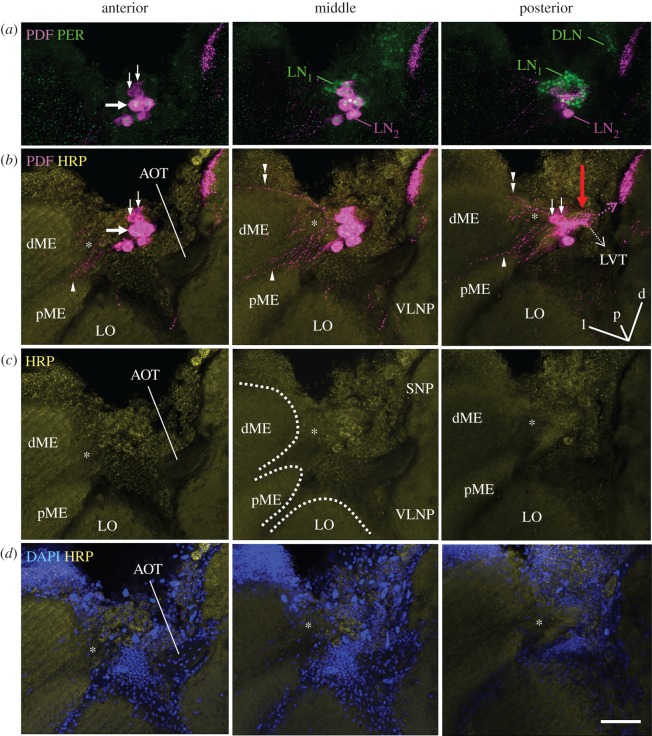
Fibres arising from the PDF-positive somata towards the medulla and the protocerebrum. All pictures are from the same 60 µm-thick semi-frontal vibratome section that is tilted in the anterior–posterior as well as in the left–right plane to visualize the serpentine layer of the medulla (the orientation is indicated in the posterior plane of (*b*); orientation arrows: l, lateral; p, posterior; d, dorsal). Each picture shows an overlay of six confocal stacks in the anterior, middle and posterior area around PDF-positive somata, respectively. The vibratome section is labelled with anti-PER, anti-PDF, anti-HRP and DAPI. (*a*) PER and PDF labelling to demonstrate the vicinity of the LN_1_ and LN_2_ (compare figures 1*a* and 3). All LN_2_ cells contain PDF and PER. DLN dorsolateral clock neurons. PDF-positive neurites arising from the LN_2_ are hard to see, because we reduced PDF labelling intensity. The large arrow points to the largest PDF neuron, the small arrows to the smallest PDF neurons. (*b*) PDF labelling at higher intensity in order to reveal the arborizations of the LN_2_ on top of HRP labelling. HRP visualizes the major neuropil regions (plus the somata of neurons): dME, distal medulla; pME, proximal medulla; LO, lobula; SNP, superior neuropils; VLNP, ventrolateral neuropils. The PDF neurons send sparse neurites into a small neuropil reminding the accessory medulla of other insects (asterisks) and from there into the serpentine layer of the medulla (arrowhead) and to a minor degree into the most distal layer of the medulla (double arrowhead). In the direction towards the protocerebrum the PDF neurons send many fibres with dense varicosities onto the surface of the lobula (red arrow) and from there to the SNP (magenta broken arrow). From the densely labelled fibres anterior of the lobula, the lobula valley tract (LVT) originates and projects posteriorly and ventrally along the inner surface of the lobula (broken white arrow; for details on the LVT figure 4). Please note that the AOT is free of PDF fibres. (*c*) HRP labelling alone. The borders of the distal and proximal medulla (dME and pME) and the LO are indicated by broken white lines in the middle picture. The serpentine layer, in which the PDF neurons project (see (*b*)) is located between the dME and pME. (*d*) DAPI and HRP labelling. The neuropils marked by HRP are free of somata and nuclei. Large tracts such as the AOT usually contain the nuclei of glia cells, whereas the putative accessory medulla (asterisk) is basically free of nuclei. Scale bar, 50 µm.

**Figure 3.**
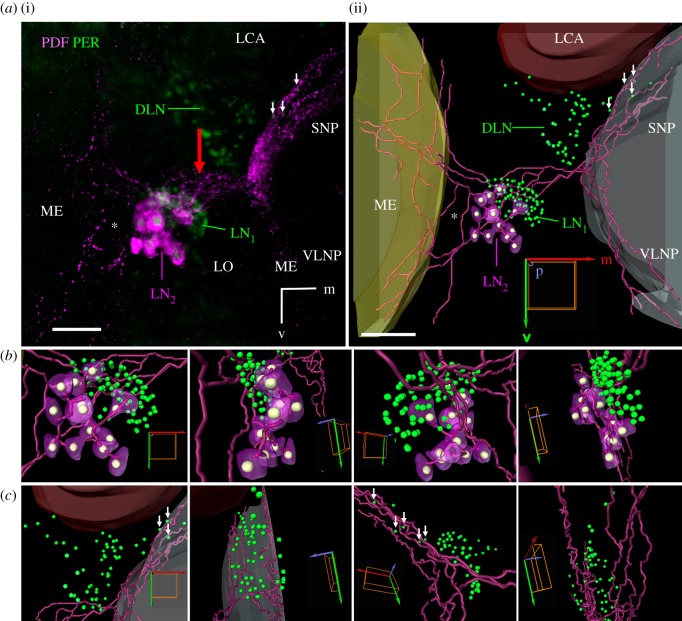
Vicinity of the PER-positive LN_1_ and DLN clusters to PDF-positive fibres originating from the LN_2_ cluster. (*a*) Projection picture of several layers scanned though the left lateral part of a whole-mount honey bee brain (i) and relevant reconstruction of this part in Amira (ii). PDF (magenta) is present in the cytoplasm of the LN_2_ cluster and labels additionally its neurites, whereas PER (green) labels the nuclei of all three cell clusters (DLN, LN_1_ and LN_2_). The nuclei of the LN_2_ cluster are shown in white in the reconstruction. Note that the PDF neurites running to the medulla (ME) are less dense than the PDF neurites running towards the superior (SNP) and ventrolateral neuropils (VLNP). The LVT is not yet visible at this anterior level. The PDF-positive neurites from the LN_2_ at the surface of the lobula (red arrow) pass the LN_1_ in close proximity. Furthermore, the ones running into the SNP are close to a few DLN neurons (small white arrows). (*b*) Larger magnification of the reconstructed LN and (*c*) DLN clusters viewed from anterior, lateral, posterior and medial, respectively. Scale bars, 50 µm. LCA, lateral calyx; orientation arrows: m, medial, p, posterior, v, ventral.

**Figure 4.**
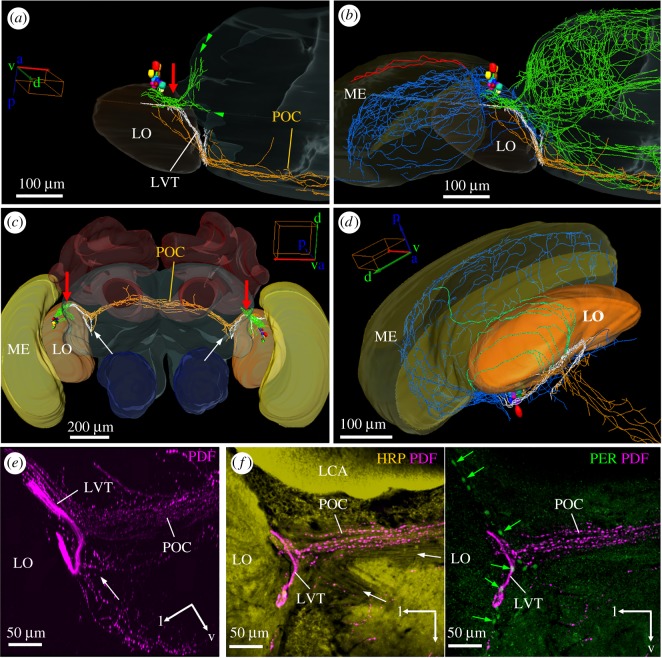
The PDF-positive lobula valley tract (LVT) and the main fibre tracts arising from it. (*a*) Reconstruction of the PDF somata and the fibres arising from them from a dorsal view of the left brain hemisphere, medulla (ME) and lobula (LO). The LVT (white fibres) originates from the dense PDF fibres on the anterior surface of the LO (red arrow) and runs posteriorly and ventrally always remaining on the surface of the LO. On its way, many fibres leave (or enter) the LVT; fibres towards the anterior surface of the protocerebrum (green double arrowhead; e.g. the SNP and VLNP as shown in figure 3), fibres towards the medial protocerebrum (green single arrowhead), fibres towards the LO (orange) and fibres to the posterior optic commissure (POC; orange). (*a*) Shows only the main origin of the fibres running towards the protocerebrum, whereas the (*b*) shows all fibres in the protocerebrum (green). In addition, it shows the fibres in the serpentine layer of the medulla (blue) and those on its distal surface that innervate the lamina (red). (*c*) Gives an overview on the course of the POC from an anterior view. In addition, the characteristic loop of the LVT at the posterior rim of the LO can be seen (white arrows). The PDF fibres anterior of the LO, from which the LVT starts, are again marked by red arrows. (*d*) is a magnification of the LVT course showing its characteristic loop and additional fibres (green) that originate from it and run distally along the posterior surface of the LO. (*e,f*) Overlay of several confocal pictures depicting the LVT at its characteristic loop plus PDF-positive fibres in the POC and in a more ventral tract that seems to start close to the loop (white arrow). Please note the many varicosities in the POC and the other tract, whereas the LVT contains virtually no varicosities in this place. (*e*) Shows only PDF labelling (overlay of six confocal stacks of a whole-mount brain). (*f*) Vibratome section labelled with PDF, HRP and PER (overlay of five confocal stacks). The overlay of HRP and PDF staining shows other fibre tracts in addition to the POC (white arrows). The right picture shows PDF and PER overlaid. PER is present in several glia cells (no HRP present in their cytoplasm) that are aligned at the border of the LO (green arrows) and at the origin of the POC.

**Figure 5.**
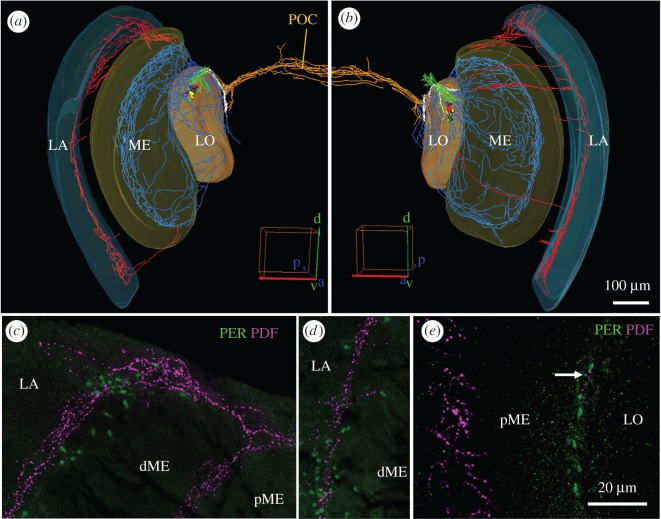
PDF fibres in the medulla and lamina. The upper row shows reconstructions of both optic lobes. (*a*) PDF fibres running to the lamina (LA) are mainly present in the dorsal medulla (ME) and to a lesser extent in the ventral ME. (*b*) The fibres running to the LA are also most prominent in the dorsal optic lobe, but in addition, fibres projection in an anterior fan over the distal surface of the ME to the LA is more frequent. For better distinction, PDF fibres invading the LA are shown in red, those invading the ME in blue, those in the dense arborization area in front of the lobula (LO) in green, those in the lobula valley tract in white and those in the posterior optic commissure (POC) in orange. (*c–e*) Magnifications of PER/PDF double labelled vibratome sections in the optic lobes. (*c*) Dorsal part of the LA and ME (overlay of six confocal stacks). Note that the fibres entering the proximal LA are in close vicinity to PER-positive glia cells. The ME is divided into proximal (pME) and distal (dME) part, which are separated by PDF-positive fibres in the serpentine layer. In the serpentine layer only few PER-positive glia cells are located. (*d*) The PDF fibres in the more ventral part of the LA are also accompanied by PER-positive glia cells (overlay of six confocal stacks). (*e*) PER-positive putative glia cells between ME and LO (overlay of three confocal stacks). In this region only sparse PDF innervations are found (white arrow).

On their way towards the central brain, all PDF neurons project into a high-density network located in front of the lobula (red arrows in figures 2–4) and less intensively into an accessory medulla that should be located at the base of the medulla. To localize the accessory medulla in the bee brain, we used HRP and DAPI counterstaining which mark the neuronal membrane and cell nuclei, respectively. With this approach, we identified an area at the base of the medulla that was free of nuclei and innervated by PDF fibres (figure 2). The latter continued into the serpentine layer of the medulla (arrowhead in figure 2; blue fibres in figures 4 and 5) and to a minor degree into the most distal layer of the medulla and into the lamina (double arrowhead in figure 2; red fibres in figures 4 and 5). Nevertheless, the innervation of this area by PDF fibres was not very prominent, but of similar density as the innervation of the medulla.

#### 3.2.2. Vicinity of Pigment-Dispersing Factor-positive fibres and the protein Period-positive LN_1_ and dorsolateral neurons

PDF fibres that run towards the central brain pass through the PER-positive LN_1_ cluster and seem to touch many of these cells (figures 2 and 3*a*,*b*). Subsequently, the PDF-positive fibres run towards the central brain along the anterior surface of the lobula. At the proximal anterior rim of the lobula several PDF fibres leave the network and project to the superior and, to a minor degree, also to the ventrolateral neuropils of the anterior protocerebrum (figure 3; green double arrowhead in figure 4*a*). On their way, the PDF-positive fibres running to the superior neuropils pass by the dorsomedial part of PER-positive DLNs cluster, in which they appear to be in close proximity to a subset of these neurons (white arrows in figure 3*a*,*c*).

#### 3.2.3. The lobula valley tract

The majority of PDF fibres do not leave the surface of the lobula but turn posterior and join a compact tract that continues to run along the surface of the lobula until it reaches the lobula's ventral posterior rim (white fibre tract in figure 4). This conspicuous tract probably corresponds to the lobula valley tract that was described in detail for the cockroach and that serves as a kind of ‘highway’ connecting clock centres in the accessory medulla with the protocerebrum as well as the ipsi- and contralateral accessory medulla [51]. This tract seems to play a similar role in the honey bee because most if not all PDF fibres seem to canalize in the lobula valley tract. They seem to enter the lobula valley tract from different brain regions and to leave it at various places. For example, on the way of the lobula valley tract to the posterior lobula many PDF fibres leave or enter it (green fibres in figure 4). The latter run on the posterior surface of the lobula and connect the lobula with the serpentine layer of the medulla (the green fibres meet the blue fibres in figure 4*d*). We do not know whether these fibres leave the serpentine layer of the medulla and run over the posterior surface of the lobula into the lobula valley tract, or *vice versa*. The same is true for all other fibres that originate from the lobula valley tract: they may enter or leave the lobula valley tract. After having reached the posterior rim of the lobula the honey bee lobula valley tract makes a characteristic loop and turns back to the anterior lobula (figure 4*a*,*b*). Then it ‘dissolves’ in many small fibres innervating the protocerebrum (green in figure 4*b*).

Just before the lobula valley tract turns back anteriorly, several fibres leave it and enter the posterior optic commissure (POC) or, *vice versa*, fibres coming from the contralateral hemisphere leave the posterior optic commissure (orange fibres in figure 4*c*,*d*) and enter the lobula valley tract close to its loop. Other PDF-positive fibres leave or enter the lobula valley tract loop from fibre bundles running to, or coming from, ventral parts of the protocerebrum (figure 4*e*). The ‘loop’ of the lobula valley tract can be easily discerned in posterior vibratome or confocal sections, and therefore we later quantified PDF staining intensity in it. Most interestingly, PER-positive glia cells are aligned at the posterior rim of the lobula and some of them seem to be in close proximity of the PDF-positive fibres in this place (figure 4*f*).

#### 3.2.4. Pigment-Dispersing Factor-positive fibres extending into the lamina

The fibres running in the distal layer of the medulla (fibres marked by a double arrowhead in figure 2; red fibres in figure 4) do not remain in the medulla, but extend into the lamina (figure 5). Most of them leave the medulla dorsally (in its dorsal rim area), as can be seen best in the left optic lobe shown in figure 5*a*. This optic lobe is an exceptional case, in which PDF-positive fibres running in the distal layer of the medulla are only present at the dorsal and ventral rim of the medulla. Usually, the PDF fibres form in addition a loose fan of distally projecting PDF fibres that extends over the entire surface of the medulla (right optic lobe shown in figure 5*b*). This loose fan innervates mainly the proximal lamina, leaving the distal lamina devoid of PDF. Only in the most dorsal region (dorsal rim region of the lamina) some PDF fibres extend more distally (figure 5*c*). This is also the region, in which the PDF fibre network between the medulla and lamina is rather dense and many PER-positive glia cells are located (figure 5*c*). A similar close vicinity between PDF fibres and PER-positive glia cells exists in the more ventral part between medulla and lamina (figure 5*d*), as well as between lobula and medulla (figure 5*e*), although in the latter area, the PDF network is not very dense.

#### 3.2.5. Pigment-Dispersing Factor-positive fibres in the proto- and deuto- and tritocerebrum

**Figure 6.**
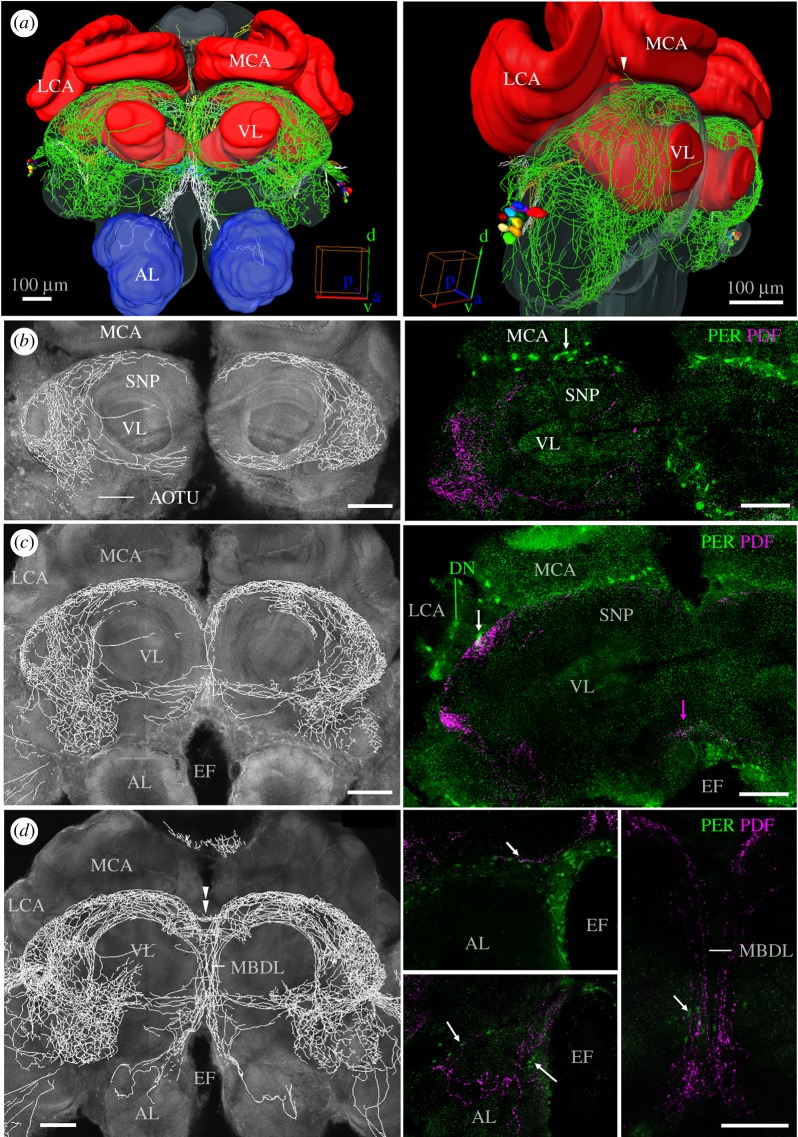
PDF fibres and PER-positive cells in the proto- and deutocerebrum. (*a*) The PDF fibres form a network on the entire surface of the protocerebrum (green fibres) without penetrating the mushroom bodies. The fibres travel through the median bundle (MBDL in *d*) ventrally to the ALs of the deutocerebrum. The medial (MCA) and lateral calyces (LCA) as well as the vertical lobes (VL) of the mushroom bodies are embraced but not entered by PDF fibres. The same applies for the AOTUs (*b*). (*b–d*) Frontal optical sections of the brain from anterior to posterior. The pictures on the left side represent reconstructions of the PDF fibres that are overlaid on the neuropil staining; the pictures on the right are confocal pictures of vibratome sections labelled for PER and PDF at a corresponding depth. Most anteriorly (*b*), PER-positive putative glia cells (white arrow) are aligned in a row between the MCA and the superior neuropils (SNP). A bit deeper in the brain (*c*), PDF fibres running underneath the LCA and MCA touch PER-positive putative glia cells (white arrow). Other PDF fibres (not seen here) come close to the PER-positive DN. In the ventral protocerebrum just ventrally of the VL, PDF fibres cross the midline of the brain in an anterior ventral commissure (magenta arrow). EF, oesophageal foramen. Still deeper in the brain (*d*), PDF fibres cross the midline in an anterior dorsal commissure (double arrowhead), others run ventrally in the median bundle (MBDL). PDF fibres in the MDBL that are accompanied by PER-positive putative glia cells (white arrow in the picture to the most right) extend ventrally into the dorsal AL (middle lower picture). Again, they are close to PER-positive glia cells (white arrows). At a slightly more anterior level (upper middle picture), PDF-positive fibres run together with PER-positive glia cells along the ventral border of the protocerebrum (white arrow). Scale bars, 100 µm.

**Figure 7.**
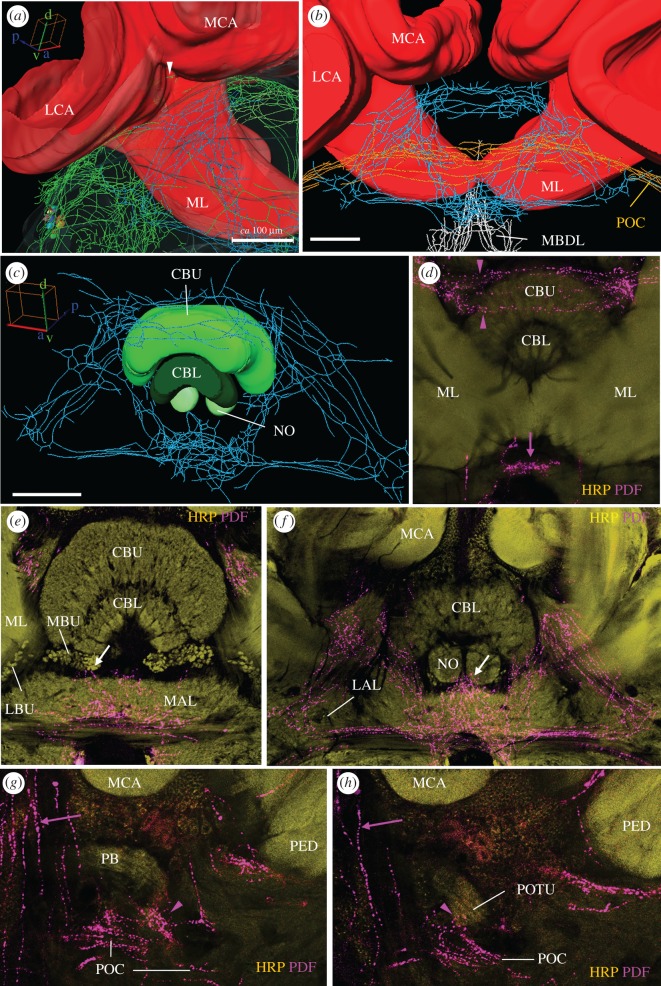
PDF fibres and PER-positive cells posterior of the mushroom bodies, around the central complex and in the posterior optic tubercles. (*a,b*) PDF fibres posterior of the mushroom bodies. The white arrowhead marks the two PDF fibres that pass between the lateral and medial calyx (LCA and MCA) from the anterior to the posterior protocerebrum. The fibres that compose the conspicuous three-triangle network just posterior of the medial lobes (ML) are shown in blue, the ones of the posterior optic commissure in orange (POC); fibres in the anteriorly located median bundle (MBDL) are depicted in white. (*c*) Central complex with the upper and lower division of the central body (CBU and CBL) and the noduli (NO) from anterior. The protocerebral bridge is omitted. PDF fibres crossing the midline in two commissures loosely wrap the CBU. (*d*) PDF fibres in the dorsal (magenta arrow heads) and ventral (magenta arrow) double commissures at the most anterior level of the mushroom bodies at which the two MLs touch each other in the midline of the protocerebrum. (*e,f*) PDF fibres at the levels of the central and lateral complex, respectively. (*e*) PDF fibres arborize in the medial accessory lobe (MAL) and extend to the medial bulbs (MBU) of the lateral complex that are characterized by a glomerular structure (white arrow). The lateral bulbs (LBUs) of the lateral complex are free of PDF fibres. (*f*) The triangles formed by PDF fibres and described in the text. The white arrow points to PDF fibres that touch the noduli. (*g,h*) PDF fibres running behind the triangle network. The magenta arrows points to PDF fibres leaving the POC and projecting to the ocelli (see also electronic supplementary material, figure S6). (*g*) PDF fibres leaving the POC terminate close to the posterior part of the protocerebral bridge (PB) (magenta arrowhead). (*h*) The right posterior optic tubercle (POTU) is innervated by PDF fibres (magenta arrowhead) stemming from the POC. All five confocal pictures are overlays of 3 confocal stacks. PED, peduncle. Scale bars, 100 µm.

**Figure 8.**
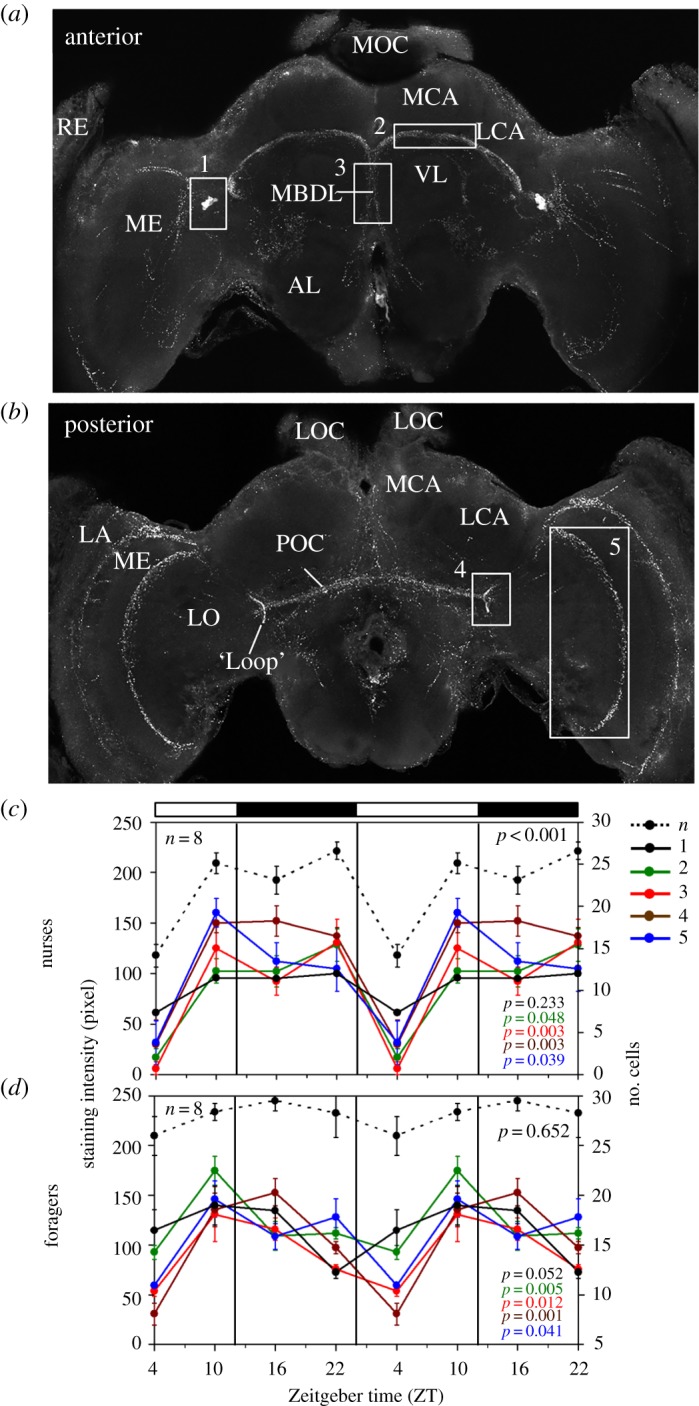
PDF-immunostaining intensity over the day in nurses and foragers. (*a*,*b*) show overlays of confocal images in the anterior and posterior honey bee brain. The numbered boxes demarcate the brain areas in which we measured PDF staining intensity (rectangles 1–5). RE, retina; LA, lamina; ME, medulla; LO, lobula; MOC and LOC, medial and lateral ocelli; MCA and LCA, medial and lateral calyx of the mushroom body; AL, antennal lobe; POC, posterior optic commissure; MBDL, median bundle; VL, vertical lobe. (*c*,*d*) Summarizes our records of PDF staining intensity for nurses and foragers sampled every 6 h from an observation hive*.* Different colours correspond to different brain areas as shown in the upper panels and in the legend to the right: *n*, number of PDF-positive somata; 1, staining intensity of the somata; 2, staining intensity of the fibres between MCA and VL; 3, staining intensity in the MDBL; 4, staining intensity in the loop of the lobula valley tract; 5, staining intensity in the posterior serpentine layer of the ME. The bars on top show the LD illumination regime, with white bars indicating light and black bars indicating darkness. For clarity the results are double plotted. Immunostaining intensity is indicated on the left *y*-axis and the relevant curves are shown as continuous lines in the diagrams. The number of stained PDF neurons is indicated on the right axis and shown as stippled black line in both diagrams. Summary of the Time effect in two-way analyses of variance (ANOVA) is shown in the bottom-right corner of each plot. The colours of the *p*-values correspond to the legend. Numbers of evaluated animals are given in the diagrams.

Most of the PDF fibres entering the protocerebrum form a dense network at its surface (green fibres in figure 6*a*). This network surrounds the mushroom bodies, but does not invade them (see also figure 7). A specifically dense PDF fibre network is present in the dorsal protocerebrum between the vertical lobes and the calyces of the mushroom bodies. This fibre network extends extremely close to the calyces of the mushroom bodies. We later determined the intensity of PDF staining in the main fibre bundle below the calyces that continues into the median bundle (see rectangle no. 2 in figure 8). Between the lateral and medial calyces, two individual PDF fibres leave this main bundle at the level of the PER-positive dorsal neurons (DNs) (white arrowhead in figure 6*a*) and cross between the lateral and medial calyx towards the posterior protocerebrum (white arrowhead in figure 7). In the anterior area between the lateral and medial calyx, the PDF fibres come close to the DN, (figure 6c) and particularly to PER-positive glia cells that are aligned in a row between the dorsal protocerebrum and the calyces (white arrows in figure 6*b*,*c*; electronic supplementary material, figure S2).

Medially, some fibres running between the calyces and the vertical lobes of the mushroom bodies extend into the median bundle (figure 6*d*). Others form an anterior dorsal commissure that projects to the contralateral brain hemisphere (figure 1, commissure 1, figure 6*d*, double arrowhead; see also below). According to its location this commissure might be homologous to the anterior optic commissure described in the cockroach [51]. In addition, we found a close relationship between PDF-positive fibres and PER-positive glia cells in the median bundle (figure 6*d*). Some fibres of the median bundle continue ventrally and invade the deutocerebrum (i.e. the dorsal part of the ALs from posterior; white fibres in figure 6*a*). The deutocerebral PDF fibres are again loosely accompanied by PER-positive glia cells and arborize between the dorsal glomeruli of the ALs (figure 6*d*). They do not seem to enter the glomeruli. Furthermore, in the ventral protocerebrum, just above the deutocerebrum, PDF fibres are close to PER-positive glia cells that are aligned in a row (white arrow in figure 6*d*). PDF fibres do also invade the tritocerebrum: posterior to the ALs, they surround the oesophageal foramen, again closely accompanied by PER-positive glia cells (electronic supplementary material, figure S3).

The anterior optic tubercles (AOTUs) (figure 6*b*) are surrounded by a dense PDF fibre network, but fibres do not enter them (electronic supplementary material, figure S4). The AOTUs of each brain hemisphere receive information from the compound eye via fibres from the lobula and medulla running in the anterior optic tract (AOT). The AOT does also not contain any PDF-positive neurites (see HRP/PDF staining in figure 2). Thus, a major light input pathway to the protocerebrum is free of PDF fibres. Nevertheless, we found PDF in a second light input pathway to the protocerebrum, the posterior optic tubercles (see below). Ventrally of the vertical mushroom body lobes, PDF-positive neurites extend towards the median protocerebrum and form a second anterior commissure (figure 6*c*,*d*; figure 1, commissure 7).

#### 3.2.6. Pigment-Dispersing Factor-positive fibres in the middle of the protocerebrum surround the mushroom bodies and the central complex

The PDF fibres in the medial brain stem partly from the PDF network on the surface of the anterior brain shown in figure 6 and partly from PDF fibres that leave the lobula valley tract on its way to the posterior rim of the lobula and enter the medial brain (see green arrowhead in figure 4*a*). Some of these fibres surround the vertical lobe and the peduncle of the mushroom body in a ring-like fashion (electronic supplementary material, figure S5). Others form a conspicuous trapezoid network made of three triangles (two side ones with their tip up, and one with an opposite orientation connecting the two) behind the medial lobes of the mushroom bodies (figure 7*a*,*b*) and around the central complex (figure 7*c*–*f*). Part of this three-triangle network are two double commissures (figure 7*c*,*d*), which arborize broadly anterior and posterior of the dorsal and ventral edges of the central complex, respectively (figure 1, commissures 2, 3 and 5, 6). The dorsal double commissure (figure 1, commissure 2, 3) runs anterior and posterior of the upper unit of the central body (figure 7*d*). The ventral double commissure (figure 1, commissure 5, 6) is part of the base side of the three-triangle network (figure 7*b*,*c*).

None of the PDF fibres in the three-triangle network enter the central complex, but the fibres in its ventral part have a close relation to the lateral complex, neuropils that are associated with the central complex [52]. The lateral complex consists of the medial and lateral accessory lobes and the medial and lateral bulbs [53]. Many PDF fibres running in the ventral double-commissure of the triangle network leave it in the middle and invade the medial accessory lobe, which is located directly underneath the lower unit of the central body and the medial bulbs (figure 7*e*). Anteriorly, these fibres appear to touch the medial bulbs of the lateral complex (white arrow in figure 7*e*) and, posteriorly, they seem to extend to the noduli (arrow in figure 7*f*), although they do not invade them. Furthermore, the lateral sides of the two triangles appear associated with the lateral accessory lobes (figure 7*f*). Throughout the three-triangle network PER-positive glia cells accompany the PDF-positive fibres in this area (electronic supplementary material, figure S3).

Posterior of the two double commissures runs the posterior optic commissure, which connects the PDF neurons of the two hemispheres (orange fibres in figures 4, 5, 7). Several PDF fibres leave the medial accessory lobe and the posterior optic commissure, project dorsally and invade the ocelli (figure 7g,*h*; electronic supplementary material, figure S6).

#### 3.2.7. Pigment-Dispersing Factor-positive fibres in the posterior optic tubercles

The posterior optic tubercles are small neuropils located in the posterior brain, adjacent to the protocerebral bridge and the posterior optic commissure [9,54]. In locusts they are part of a potential second polarization vision pathway that runs in the posterior optic commissure and arborizes in the accessory medulla [55]. In cockroaches and locusts they are invaded by PDF fibres arborizing in the accessory medulla and leaving the posterior optic commissure [9,51]. In the honey bee, the posterior optic tubercles are less well characterized, but we found similar fibres that leave the posterior optic commissure and arborize in these small neuropils (figure 7*g*). Furthermore, some PDF fibres leaving the posterior optic commissure project in the direct vicinity of the protocerebral bridge (figure 7*h*).

### 3.3. Temporal variation in Pigment-Dispersing Factor immunostaining intensity

If PDF is involved in transmitting time-of-day information, one would expect it to be rhythmically released into target brain areas. We tested this hypothesis by measuring PDF-immunostaining intensity throughout the day in the following regions of interest: (1) the PDF neuron somata (figure 8, rectangle no. 1), (2) the area ventrally to the calyces of the mushroom bodies (figure 8, rectangle no. 2), (3) the median bundle (figure 8, rectangle no. 3), (4) the lobula valley tract at its most posterior location, the ‘loop’ (figure 8, rectangle no. 4) and (5) the posterior rim of the serpentine layer in the medulla (figure 8, rectangle no. 5). Changes in staining intensity may be due to peptide release or differences in levels in the cell. In addition, changes in neuron structure are possible [28]. We were not able to detect such structural changes in our preparations (electronic supplementary material, figure S7). Thus, the changes we report below appear to stem mainly from temporal variation in PDF levels.

#### 3.3.1. Pigment-Dispersing Factor oscillations in nurses and foragers under entrained conditions

The first two experiments compared PDF-immunostaining intensity for forager and nurse bees from two different source colonies in Israel. We found time-dependent changes in PDF-staining intensity in both foragers and nurses: PDF-staining intensity was lowest at the beginning of the day and highest at the end of the day/beginning of the night. In the first experiment that included four time points over a single day, there was a significant influence of time of day on PDF-immunostaining intensity in all the focal arborization areas (*p* < 0.05; figure 8). Furthermore, we revealed a significant PDF cycling in the cell bodies of foragers and a significant oscillation in the number of immunostained cell bodies in nurses (figure 8). An overall very similar temporal pattern was obtained in the second experiment in which bees were sampled over six time points per day. However, in this experiment only the variation for the lobula valley tract fibres of foragers and the fibres ventrally of the calyces of nurse bees were statistically significant at the *p* < 0.05 level by ANOVA and the JTK_CYCLE analysis. It should be noted that this experiment had lower statistical power because sample size was only three to four brains per time point (electronic supplementary material, figure S8).

Taken together, these two experiments point to a possible difference between oscillations in the PDF network of nurses and foragers: in nurses, the oscillations in the five areas of interest showed high synchrony (correlation coefficient (*r*) between staining intensity in the PDF cell bodies and the different fibres was between 0.854 and 0.992 in the first experiment, and between 0.871 and 0.932 in the second experiment). All the measured PDF fibres show a sharp drop in staining intensity at the early morning (figure 8; electronic supplementary material, figure S8). In foragers this drop appeared more gradual and overall PDF cycling was less synchronous among the focal areas (0.027 < *r* < 0.653 in the first experiment and 0.208 < *r* < 0.449 in the second experiment) when compared with nurses (figure 8; electronic supplementary material, figure S8).

#### 3.3.2. Pigment-Dispersing Factor oscillations in foragers under constant dark conditions

**Figure 9.**
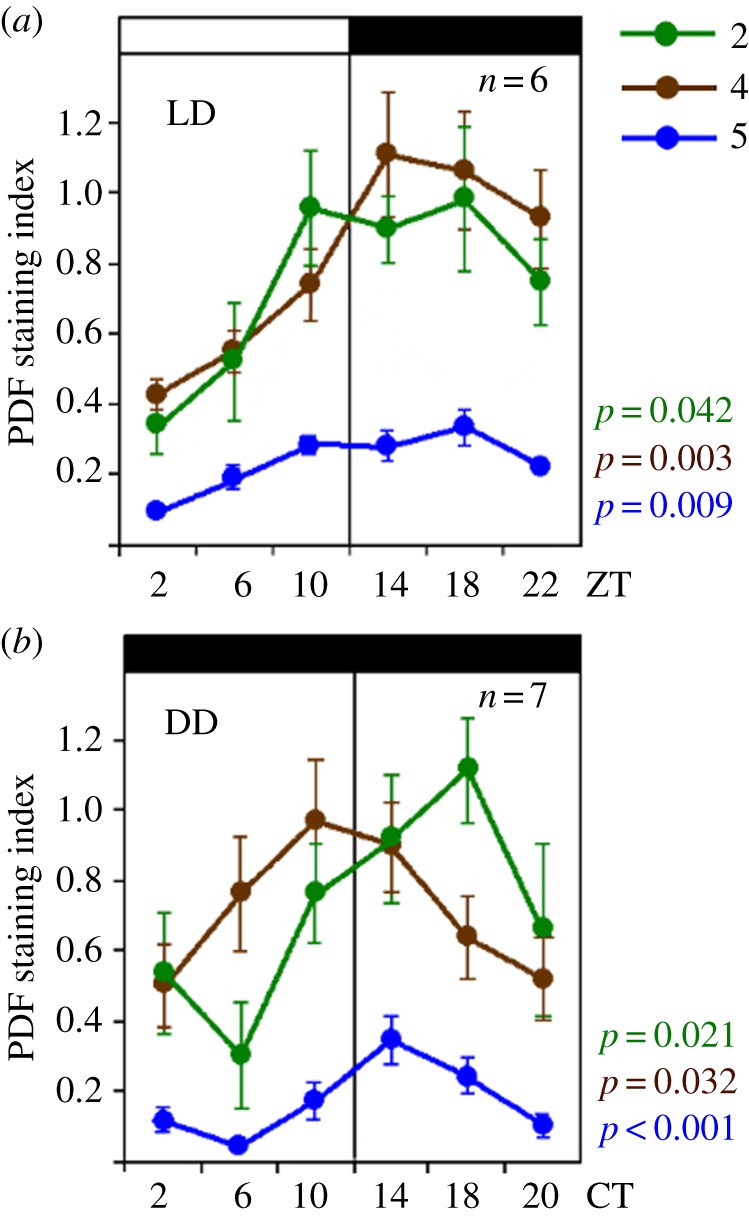
PDF-immunostaining intensity over the day in foragers sampled in LD and DD illumination regimes. PDF staining intensity was evaluated at six different times in (*a*) LD 12 : 12 (*n* = 6 bees per time point) and (*b*) on the fourth day in constant darkness (DD; *n* = 7). Note that the *y*-axis depicts the PDF staining index, the scale of which is different from the staining intensity in figure 8. This is because the PDF staining intensity was evaluated by a different method [29] (see Material and methods). ZT, Zeitgeber time; CT, circadian time. Other details are similar to figure 8, except that the given *p*-values for DD are calculated with the JTK_CYCLE analysis rather than with ANOVA (see text for details).

This experiment was performed with bees collected in Germany. We quantified PDF staining intensity only in the fibres between the vertical lobes and calyces, in the lobula valley tract and in the serpentine layer of the medulla (rectangles no. 2, no. 4 and no. 5 in figure 8, respectively). We first repeated the six-time-point experiment in LD 12 : 12 (this time under artificial light in an incubator) to compare the pattern of PDF levels over the day in bees from Germany and Israel. Although PDF-immunostaining intensity in the fibres in this experiment was higher than in the two experiments with bees shipped from Israel, the temporal patterns were very similar: PDF staining in all areas of interest was higher during the night than during the day with a clear minimum occurring at the beginning of the day (ZT2; figure 9). An ANOVA revealed significant time-dependent differences in all focal PDF fibres areas (*p* < 0.05). Furthermore, the variation over the day in the different PDF fibres was again slightly out of phase, as revealed by rather low correlation coefficients between the three areas of interest (0.10 < *r* < 0.38).

Next, we assessed PDF levels in foragers sampled under constant darkness and temperature over a single day, thus, representing genuine endogenous rhythms. We sampled foragers and kept them for 3 days in a constantly dark incubator. We processed samples of bees collected every four hours for PDF-immunocytochemistry on the fourth day in DD. The results presented in the lower panel of figure 9 show PDF-immunostaining intensity throughout one cycle in CT. To estimate CT, we assumed that the bees are free-running with a period of 23.5 h (conferring to the free-running periods measured previously for bees from a comparable German apiary [56]) and accordingly treated each day as lasting 23.5 rather than 24 h (i.e. CT 24 is actually 23.5 h after CT0). Indeed, visual inspection of the curves reveals oscillations in PDF staining intensity with the expected phase: in all three areas of interests, PDF staining intensity appeared higher during the subjective night than during the subjective day (figure 9). ANOVA revealed a statistically significant effect of time only for the serpentine layer of the medulla (blue line in figure 9; *p* = 0.01). However, in a complementary non-parametric JTK_CYCLE analysis we found statistically significant oscillations in all three areas of interest (*p*-values in figure 9).

### 3.4. The influence of Pigment-Dispersing Factor injection into the brain on circadian rhythms in locomotor activity

To further test the hypothesis that PDF signalling is important for circadian rhythms in honey bees, we tested the influence of PDF injection on circadian rhythms in locomotor activity. We used two different protocols to inject PDF at CT 8 to CT11 into one side of the brain, laterally to the PDF-positive somata.

#### 3.4.1. Injections after cutting a window in the head capsule cuticle

**Figure 10.**
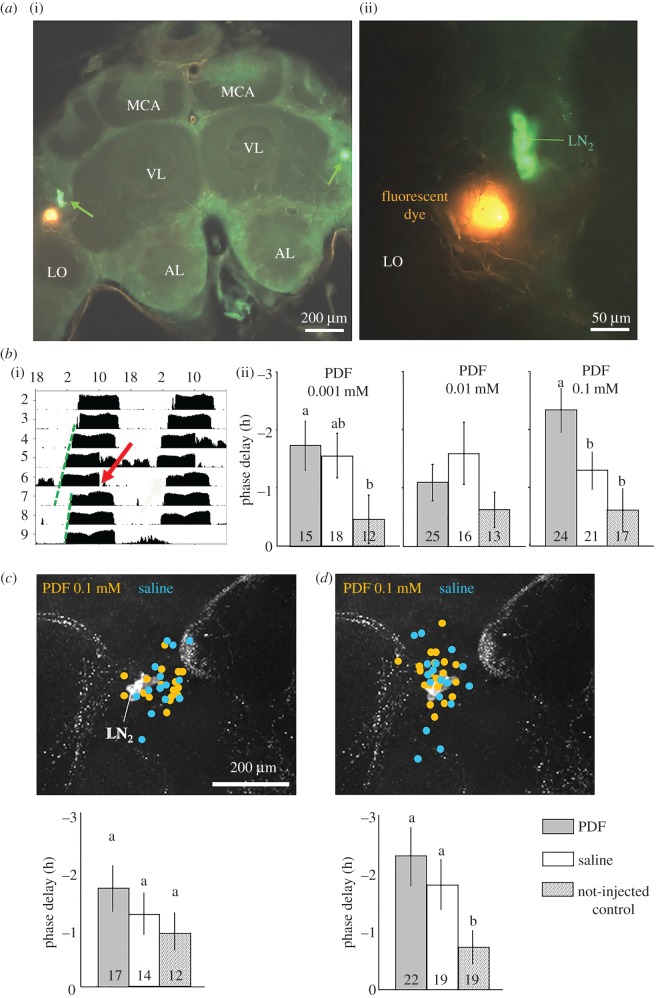
PDF and saline injection into the brain close to the PDF neurons phase delays circadian rhythms in locomotor activity. (*a*) The site of injection relative to PDF neurons (=LN_2_; green arrow in left picture). (i) Overview on the honey bee brain unilaterally injected with fluorescent dye (orange) and immunostained for PDF (green). (ii) Close-up view showing that the injected die (orange) is found near LN_2_ somata, but does not appear to harm them. AL, antennal lobe; MCA, medial calyx of the mushroom body; LO, lobula; VL, vertical lobe of the mushroom body. (*b*) (i) A representative actogram of a honey bee forager injected with 0.1 mM PDF peptide. The red arrow points to the time of injection. The green dashed lines represent the calculated linear regression lines through the daily onsets of locomotor activity before and after the time of injections. The bee phase delays its activity onset upon PDF injection. (ii) The phase delays (±s.d.) obtained by PDF injections of three different doses. Every column depicts the pooled data of three trials (pooled sample size inside the bars). Only a dose of 0.1 mM PDF gave a response that was significantly different from the two controls (bars with different letters within the same plot differ statistically; two-way ANOVA with treatment (i.e. saline or PDF amount) and trial as factors, LSD post hoc test, *p* < 0.05). (*c*) Injection site and phase delays found in the first experiment, in which the injections were done through the cuticle without opening the head capsule. The injection sites were verified after the experiment and are shown as yellow and blue dots on a PDF stained brain. In this experiment, most injection sites were between the LN_2_ and the superior lateral protocerebrum (SLP). The PDF injections provoked non-significant delays. (*d*) Injection site and phase delays found in the second experiment using the same method. This time, most injection sites were very close to the LN_2_ (and the LN_1_ that lie just dorsally of the LN2; figure 3). The injections provoked large phase delays without significant differences between PDF and saline.

In the first sets of experiments, we opened a window in the head capsule cuticle, and injected PDF or saline into one optic lobe, laterally to the PDF-positive somata. A similar injection of a fluorescent dye confirmed that the injection site was indeed laterally of the PDF-positive somata (50 ± 20 µm lateral of the somata; figure 10*a*). The injection did not hit the PDF-positive somata. We then used the same procedure to inject three PDF concentrations (0.001, 0.01 and 0.01 mM) and repeated each experiment three times. Figure 10*b* shows the pooled data from all three experiments. Control bees that were similarly handled and chilled, but not injected showed a small phase delay of less than an hour on average. The phase delay was longer than 1 h for the saline and PDF injected bees. Injections of 0.1 mM and 0.001 mM PDF produced a statistically significant longer phase delay relative to un-injected control bees, but only injection of 0.1 mM PDF produced a significantly larger phase delay compared with saline injection (for statistic results, see figure legend). These results suggest PDF injection at CT8–11 caused a phase delay, but that some of this effect is caused by saline injection.

The free-running period did not differ between bees injected with PDF or saline, nor those not injected in the experiments with the three PDF doses (data not shown; two-way ANOVA; experiment with PDF 0.001 mM, *p* = 0.514; PDF 0.01 mM, *p* = 0.921; PDF 0.1 mM *p* = 0.094). The strength of circadian rhythms (power) was lower after injection, even for the control non-injected bees, but this attenuation was not affected by treatment in the experiments with PDF 0.01 mM (ANOVA, *p* = 0.6) and 0.1 mM (*p* = 0.45). In the experiment with the lowest PDF dose (0.001 mM), the decrease for the control (non-injected) bees was significantly larger (data not shown; two-way ANOVA, LSD post hoc test, *p* < 0.05). Thus, we conclude that the injection of PDF or saline did not affect the strength of circadian rhythms.

#### 3.4.2. Stereotactic injections through the head capsule cuticle without cutting a window

Based on the results of the first set of experiments, we injected a concentration of 0.1 mM PDF in this second set of experiments. In the first trial, the estimated injection sites were mostly located proximally of the PDF somata, only a few may have hit the somata themselves (figure 10*c*), in the second trial they were located closer to the somata (figure 10*d*). Based on the estimated site of injection it appears that injection site affected the measured phase shift (data not shown). Although in a few bees saline or PDF injection caused a phase advance, on average the injections induced a phase delay which appears larger after injecting PDF compared to saline (but this difference was not statistically significant). In the second trial however, the PDF injection caused a significant phase delay relative to the control. In both trials using this protocol, the injection of PDF or saline did not affect the period (FRP) or strength (power) of circadian rhythms in locomotor activity (*p* > 0.05, data not shown).

## 4. Discussion

The experiments summarized above lend credence to the hypothesis that PDF neurons in the honey bee brain fulfil the required anatomical prerequisites to convey rhythmic signals to other brain regions, including those involved in time-associative memory and time-compensated sun-compass orientation. We show that PDF is most likely to be rhythmically released into diverse relevant brain regions. Our injection experiments further suggest that PDF can phase shift the circadian rhythm of locomotor activity. Importantly, most of our findings were confirmed for bees from different colonies (which are genetically different) and even for stocks in different countries, adding to the generality and robustness of our findings. These findings for an insect from the order Hymenoptera are consistent with earlier studies showing that PDF plays a pivotal role in the circadian system of the fruit fly *Drosophila melanogaster* (Diptera), the cockroach *Rhyparobia maderae* (Blattodea) and the cricket *Gryllus bimaculatus* (Orthoptera).

### 4.1. Pigment-Dispersing Factor fibres pass next to protein Period-expressing neurons and glia cells

The clock protein PER of the honey bee *A. mellifera* is present in neurons in the lateral and dorsal brain, as well as in numerous glia cells throughout the brain and the optic lobes similar to the distribution in *D. melanogaster* [20,57,58]. Here we show that fibres stemming from the PDF-positive LN_2_ come close to most other clusters of PER-expressing neurons, and are accompanied by PER-positive glia cells in most brain regions. This finding is similar to PDF-immunostaining in *Drosophila melanogaster*, in which the PDF neurites from the PDF-positive LN_v_ are accompanied by PER-positive glia cells [19,59], and come close to other PER-positive neurons in the lateral and dorsal brain [60]. In *Drosophila,* it was further shown that many of these PER-positive cells also express the PDF receptor [33,61–63] and that PDF signalling strongly affects the oscillations of the other clock neurons and the flies' behavioural rhythmicity [22,30,64–67]. Our findings that PDF levels in the honey bee brain cycle over the day, and that injection can shift the locomotor activity phase, suggest that PDF neurons have overall similar functions in honey bees and fruit flies. The close proximity of PDF fibres and PER-positive glia in the honey bee is also significant. In *Drosophila,* it was shown that electrical manipulation of PER-positive glia cells makes fruit flies arrhythmic, suggesting that glia cells are involved in the clock network and important for rhythmic behaviour [68,69]. The importance of PER-positive glia cells for circadian rhythms was also recently demonstrated in the mammalian circadian clock, the SCN [70].

### 4.2. Comparison of Pigment-Dispersing Factor arborization pattern in the honey bee brain with that of other insects

PDF immunocytochemistry was performed in the brain of many insects including locusts, cockroaches, crickets, bugs, cicadas, flies and bees [9–12,14–16,21,71–73], with the most detailed description available for the cockroach *R. maderae* (formerly *Leucophaea maderae*) [21,51,74–77] and the fruit fly *D. melanogaster* [78]. In all these species, with the exception of moths and butterflies [77], PDF-positive somata could be localized to the optic lobes or the lateral protocerebrum [79,80].

#### 4.2.1. Number and size of the Pigment-Dispersing Factor neurons

We found on average approximately 15 PDF-positive somata/brain hemisphere of different size, which are all located close to the anterior medulla. This number fits the approximately 14 PDF somata reported previously for honey bees [14,15] and the 9–15 PDF somata found in bumble bees [16]. It also roughly fits to the number of ∼12 PDF-positive somata located anteriorly of the medulla in the cockroach [74]. Whereas the cockroach possesses additionally eight PDF neurons with somata posterior of the medulla and many PDF neurons with small somata close to the dorsal and ventral lamina [51], such PDF-positive somata do not exist in the bee brain. Furthermore, whereas the anterior PDF somata of the cockroach are located at the ventromedial edge of the medulla, the honey bee PDF neurons are located at its dorsomedial edge and have a larger distance to the medulla than the anterior PDF somata of the cockroach.

In all species investigated so far the PDF somata vary in size [14,15,48,77,81,82]. We observed one particularly large, strongly stained neuron that was localized anteriorly to the other PDF-positive neurons, whereas middle-sized and smaller somata were usually located more posterior and closer to the PDF fibre network. In cockroaches and flies, the PDF neurons with large somata appear to show wide field arborizations that span the entire brain and connect both brain hemispheres [60,76,83]. In honey bees, we could not assign fibres to individual neurons, but, similar to cockroaches and flies, the large and middle-sized honey bee PDF neurons may arborize throughout the entire brain, whereas the ones with small soma may remain more local within the ipsilateral hemisphere.

#### 4.2.2. The accessory medulla looks different in the honey bee when compared with flies and orthopteran insects

The accessory medulla can be regarded as the most important communication centre in the circadian network of insects, which additionally gets input from external Zeitgebers (environmental time cues). This small neuropil at the base of the medulla is typically characterized by a dense network of PDF-positive fibres. It is best characterized in the cockroach *R. maderae*, where it is densely innervated by PDF-positive and other peptidergic neurons that may be similarly engaged in the circadian clock [74,76,77]. It is organized into a nodular core receiving photic input from the eye and into an internodular and peripheral neuropil involved in efferent output and coupling input from other clock neurons [74,77]. In *D. melanogaster*, the accessory medulla is less conspicuous, but as in the cockroach it receives dense input from the PDF-positive neurons as well as from the majority of other clock neurons and it is innervated by an extra retinal eye, the Hofbauer–Buchner eyelet [81,84,85].

We identified a small neuropil at the base of the honey bee medulla which is even less noticeable than the accessory medulla of flies and is not densely innervated by PDF-positive fibres. Interestingly, an area with a much higher density of PDF fibres was found proximally of the PDF neurons in front of the lobula. Thus, we suggest that the communication centre of the honey bee circadian clock neurons has moved from its common place in the accessory medulla into a place anterior to the lobula. A similarly located PDF-rich network was also described for the bumble bee, *Bombus terrestris* [16]. We do not know whether this new place is anatomically homologous to the accessory medulla of the cockroach or the fly. Nevertheless, the new putative circadian communication centre of bees is located very close to the somata of the PER-positive LN_1_. In *Drosophila* several lateral neurons are in close vicinity of the accessory medulla and the majority of the other PER-positive clock neurons project into the accessory medulla. Additional studies are needed in order to determine whether the bee LN_1_ and the other PER-positive clock neurons do also project into the dense PDF network anterior of the lobula.

The apparent neuroanatomical differences in the circadian clock centre of bees and other insects may be explained by developmental processes. We assume that in honey bees a close association of the accessory medulla with photoreceptors is lacking during development, and consequently a dense innervation of the accessory medulla by PDF fibres is missing. It will be interesting in the future to perform developmental studies in order to see how the PDF neurons and their arborizations look during larval and pupal stages.

### 4.3. Connections of the Pigment-Dispersing Factor neurons with higher integration centres in the brain

Rhythms in locomotor activity require that signals from the circadian clock network are transmitted to centres in the brain that modulate activity and sleep. Suited targets are peptidergic neurons in the superior protocerebrum including neurosecretory cells of the pars intercerebralis and lateralis that have been shown to modulate metabolism and activity/sleep. In addition, time memory and sun-compass navigation require that information about time of day is conveyed to brain structures responsible for memory and sun-compass orientation. Below we discuss whether PDF neurons fulfil the anatomical criteria to provide such an input.

#### 4.3.1. Connections between Pigment-Dispersing Factor neurons and brain centres that modulate the activity/sleep cycle

Neurosecretory cells in the superior protocerebrum, namely in the pars intercerebralis and lateralis have been shown to modulate metabolism, sleep, activity and other behaviours in insects [82]. In the honey bee, the somata of the lateral neurosecretory cells are distributed anterior to the peduncles of the lateral calyces, in a band that extends from the medial calyces to the ventro-lateral edge of the lateral calyces [86]. This is exactly the area where we found a very dense network of PDF fibres. Thus, it is most likely that the PDF neurons signal to lateral neurosecretory cells. A similar connection is likely between PDF fibres in the superior median protocerebrum and the neurosecretory cells of the pars intercerebralis in the bee brain. In *Drosophila melanogaster*, several output pathways from the PDF neurons to peptidergic cells in the pars lateralis and intercerebralis have been shown. (1) The PDF neurons signal on diuretic hormone 31 (DH31)-expressing neurons in the superior protocerebrum that consecutively release DH31 waking up the flies in the morning [87]. (2) In the same brain area, the PDF neurons signal to Allatostatin C-positive neurons that provoke the flies’ postprandial sleep [88]. (3) Finally, the PDF-positive neurons signal via dorsal clock neurons to diuretic hormone 44 (DH44)-positive neurons in the pars intercerebralis [89] and from there to *hugin*-expressing neurons in the suboesophageal zone that run to motor circuits in the thoracic ganglia [90]. DH44 is the homologue of the mammalian corticotropin-relasing factor that is rhythmically released from the hypothalamus to prepare the organism for action. Similarly, DH44 appears to prepare the flies for activity. It will be rewarding to investigate whether similar pathways controlling activity and sleep exist in the honey bee.

#### 4.3.2. Connections between Pigment-Dispersing Factor neurons and memory centres in the mushroom bodies

Honey bees are excellent learners, quickly forming associations between stimuli of different sensory modalities [91] and showing various forms of conceptual learning [92] that are largely mediated by their highly developed mushroom bodies. The medial and lateral calyces of the mushroom bodies are large and receive input from olfactory and visual cues [93], whereas the vertical lobes are thought to be the main output regions of the mushroom bodies [94]. We found that PDF neurons do not invade the mushroom body neuropils, but rather wrap their various parts. The densest network of PDF fibres is found ventral to the lateral and medial calyces of the mushroom bodies, but also the vertical, medial lobes and the peduncle are surrounded by varicose neurites of PDF neurons. For example, the PDF fibres form rings around the vertical lobe and the peduncle (see electronic supplementary material, figure S4). As PDF is most likely to be stored in the varicosities and released from there (and other parts of the neurite) in a paracrine manner, it can probably reach into the mushroom bodies and convey time-of-day information which can modulate cells involved in various forms of learning and memory.

#### 4.3.3. Connections between Pigment-Dispersing Factor neurons and the sun-compass pathway

The neuronal basis and mechanisms underlying sun-compass orientation have been investigated in detail in locusts (reviewed by [95,96]). Two major pathways that transfer sun-compass signals to the central complex have been described: a prominent anterior one, and a less striking posterior one [97]. Recent studies suggest that at least the anterior polarization vision pathway is conserved between honey bees, locusts, bumble bees and ants [53,98,99]. The sun-compass pathway receives skylight polarization input from a specialized area of the compound eye, the dorsal rim area. Photoreceptors in this area project through the dorsal lamina and terminate in the dorsal rim area of the medulla, which is called ‘MEDRA’. The MEDRA is innervated by transmedulla neurons that carry the polarized light information through the serpentine layer of the medulla and then via the AOT to the anterior optic tubercle [98]. From there, interneurons make a connection to the lateral and medial bulbs [98,100]. In the bulbs, they form conspicuous large synapses with GABA-ergic tangential neurons of the central body's lower division [53]. These neurons are then connected to the protocerebral bridge of the central complex which holds a topographic representation of zenithal polarization angles [101–103]. The possible posterior polarized-light input pathway starts also from the serpentine layer of the medulla and ends in the protocerebral bridge; but it passes via the accessory medulla and the posterior optic commissure to the posterior optic tubercles that locate adjacent of the most lateral endings of the protocerebral bridge. Output neurons from the protocerebral bridge project to the lateral accessory lobes [97,103,104] and, finally, polarization information is sent via descending neurons to thoracic motor control centres [105]. In honey bees, the pathways within the central complex as well as the output pathway to the lateral accessory lobes are not yet clarified, but are likely to be similar.

We found PDF-positive fibres (1) in the dorsal rim area of the lamina, (2) between the dorsal rim area of the lamina and medulla and (3) in the dorsal rim area of the medulla. These fibres extend along the projections of the dorsal rim photoreceptors that run into the MEDRA. Our results are in line with the data of Zeller *et al*. [98], who combined PDF immunocytochemistry with tracing of sky compass pathways to the MEDRA that is surrounded by PDF fibres. They further show that the PDF fibres in the serpentine layer of the medulla overlap with the pathway of the transmedulla neurons that run via the AOT into the central brain. Consistent with their observations, we do not see any PDF-positive fibres in the AOT and the anterior optic tubercle, but we see a dense PDF fibre network directly behind the anterior optic tubercle. In addition, we see a ring of PDF fibres around the vertical lobes, exactly at the location where the interneurons from the AOTUs take their path towards the medial and lateral bulbs. The bulbs themselves are not innervated by PDF fibres, but again, PDF varicosities are very close to them. We also see a dense PDF network in the medial accessory lobe, a brain area that seems to be associated with the central complex [106], and PDF fibres close to the lateral accessory lobes. The boundaries of the honey bee lateral accessory lobes are less well defined, and therefore we cannot be sure whether the PDF fibres enter them or just pass close to them. Concerning the possible posterior polarized-light input pathway, we found PDF fibres that leave the posterior optic commissure and may innervate the posterior optic tubercles. Thus, here might be a direct input from the PDF neurons.

In summary, we see a close vicinity of PDF fibres to the sky-compass pathway, not only on its input side to the central complex, but also on its putative output pathway. Thus, a rhythmic paracrine release of PDF in several places can potentially convey time-of-day information to the sun-compass network.

### 4.4. Pigment-Dispersing Factor oscillations

The transfer of time-of-day information from the LN_2_ neurons to PDF-responsive brain structures requires a time-dependent release of PDF. Indeed, we detected oscillations in PDF staining intensity in the analysed brain areas. These oscillations are consistent with the hypothesis that PDF is rhythmically released into the protocerebrum and the medulla of the bee. In all the experiments, PDF-staining intensity showed a clear trough during the early day, and highest intensity during the night. This pattern suggests that PDF is released during the early morning or subjective morning. The timing of PDF oscillation in the honey bee differs from that reported for *D. melanogaster*, in which PDF staining intensity in fibres terminating in the dorsal protocerebrum was maximal in the morning and lowest during the night [27,29]. In addition the *Drosophila* PDF terminals showed more branching in the morning, when PDF is transported to the terminals and released into the protocerebrum, than in the evening [28]. Our analyses for the honey bee are not consistent with this mechanism. PDF was always present and we did not notice significant daily changes in the varicosities and the branching, although we may have overlooked minor changes. We thus suggest that in the honey bee PDF release does not depend as much on a rhythmic transport into the terminals as it does in *Drosophila*. A more likely explanation is that PDF is stored in the varicosities, and when it is released, PDF staining intensity drops temporarily. This kind of rhythm would also fit to the rhythm in honey bee *Pdf* mRNA, which shows a maximum at the end of the day, after PDF has been released from the terminals and starts to increase again, and a minimum during the beginning of the night, when PDF levels have reached their maximum in the terminals [15]. In *Drosophila*, no cycling in *Pdf* mRNA levels has been observed [27], consistent with the premise that the PDF rhythm relies mainly on rhythmic transport of PDF into the terminals and not on rhythmic synthesis. The fact that PDF levels in *Drosophila* cycle in opposite phase in the somata and the terminals of the neurons is consistent with this premise. In the honey bee on the other hand, PDF cycling in the somata and in the fibres are in phase with each other and the observed cycle seems to depend on rhythmic synthesis and release. If our hypothesis is true, PDF would be released in the morning from the terminals of both diurnal insects.

#### 4.4.1. Pigment-Dispersing Factor levels oscillate in both foragers and nurses

Nurses are active around the clock with attenuated circadian rhythms in locomotor activity and in whole-brain *per* mRNA abundance [14]. However, some of their pacemakers generate endogenous rhythms and can be entrained by social Zeitgebers [20,107–109]. Consistent with this evidence for functional clocks, we found here that also the PDF staining intensity oscillates in the nurse brain. Nevertheless, our analyses suggest that there are some differences between nurses and foragers in the degree of synchronisation among the brain sites. In nurses, PDF oscillations in the different brain areas that we measured appear to be better synchronized, than in foragers. Although our sample size is quite limited at this stage (three colonies for foragers, and two for nurses) this finding deserves attention.

The lower synchrony in foragers may stem from their exposure to a more diverse set of external Zeitgebers (time-givers) than nurses. The nurses were directly collected from the hive and did not experience any light–dark or significant temperature cycles. Their circadian clock was probably entrained mostly by social cues (coming from the strongly rhythmic foragers) [56,108]. Another, not mutually exclusive explanation for the differences in synchrony is that the circadian system of nurses is not yet mature, and that in the mature system of foragers, PDF is released with different kinetics and at slightly different times into the diverse brain areas. Studies in which PDF oscillations are compared for nurses and foragers of a similar age can explicitly address this question.

It is too early to speculate about the biological function of this apparent task-related variability in synchrony in PDF rhythms across the brain, but rhythms with different phases are common in the brain of mammals [110]. Also in the fruit fly, clock neurons in different parts of the brain show differently phased Ca^2+^ rhythms [31,111]. Likewise, such differently phased rhythms may occur in the brain of honey bees and may be associated with the timing of PDF oscillation.

#### 4.4.2. Pigment-Dispersing Factor cycling appears to continue under constant darkness

PDF cycling in the honey bee seems to continue under constant darkness. Although ANOVA did only reveal significant effects of time-of-day in the serpentine layer of the medulla, the JTK_CYCLE analysis that tests for rhythmic patterns clearly showed a significant cycling in all three areas tested. Furthermore, it is notable that even after three days of free-run the trough in PDF staining still occurred on the early subjective morning, which is consistent with the pattern that we found in the other experiments.

### 4.5. Pigment-Dispersing Factor injections phase-shifted circadian rhythms in locomotor activity

We used two different protocols to inject PDF into the honey bee brain. With both protocols saline injection into the area between the optic lobes and the central brain caused a consistent trend of phase delay that was statistically significant in one trial (figure 10*d*). This effect is consistent with our neuroanatomical description showing that this brain area is adjacent to the LN_1_ and LN_2_ PER-positive clusters and is rich in PDF-expressing fibres that originate in the LN_2_ neurons. PDF injection caused a phase delay when injected at approximately 20–50 µm distance of the PDF neurons during the early night (using the protocol in which we opened the head capsule). We received an overall similar pattern of PDF effect that appears stronger than the saline effect with the second protocol, in which we did not open the head capsule, although the difference between PDF and saline injection was not statistically significant. We assume that this is because of the higher spatial variability of this protocol, and the fact that the PDF effect differs between sites of injection. Our findings suggesting that PDF injection phase-shifted circadian rhythms fit with similar experiments with cockroaches and crickets. Taken together the similar findings in diverse insect species, they lend credence to the hypothesis that PDF is an integral part of the circadian neuronal network in insects [34,36]. It would be interesting to know whether the PDF effect is time-dependent with a phase advance at some parts of the circadian cycle, as has been demonstrated for cockroaches and crickets. With both protocols PDF injection into one side of the brain did not affect the strength (power) or period of circadian rhythms in locomotor activity. These findings suggest that PDF injection did not modify the pace of oscillation in key pacemakers that control circadian rhythms in locomotor activity. The lack of effect on the strength of circadian rhythms suggests that the injection did not disturb the phase relationship between the various PDF-responsive neurons that are involved in the regulation of circadian rhythms in locomotor activity.

In sum, our detailed neuroanatomical descriptions reveal that PDF fibres stemming from the LN_2_ clock neurons arborize extensively in the optic lobes and central brain of the honey bee. PDF fibres reach many PER-positive neurons and glial cells, and cross to ipsilateral parts of the brain, which is consistent with a role in coupling the different components of the brain circadian network. PDF fibres are also well positioned to function in both input and output pathways of the circadian clock. These include extensive arborization in the optic lobes that integrate visual information and the dorsal rim area that is sensitive to light polarization and important for sun-compass orientation. The extensive arborizations in the central brain are in a good position to convey time-of-day information from the LN_2_ to brain centres involved in various clock output functions. These include neuropils involved in locomotor activity, sleep, sun-compass orientation and time-associative learning. The evidence that PDF levels show a clear trough during the early day (or subjective day in constant darkness) suggests that the timely release of PDF is a mechanism to convey time-of-day information to target neurons expressing the PDF receptor. This premise is further supported by our injection studies, which show that an artificial elevation of PDF levels phase shifts circadian rhythms in locomotor activity. This study together with our earlier characterization of PER immunostaining [20] provides the best description of the bee circadian network available so far, and sets the stage for studies on the interplay between the circadian clock and complex behaviours such as division of labour, dance communication, sun-compass orientation and time-memory.

## Supplementary Material

Supplemental figures and discussion

## Data accessibility

A movie showing a 360° view of the 3D PDF network reconstruction in the honey bee brain is available as electronic supplementary material (AVI file named ‘Movie PDF-network (2)’; after download it is to be opened with, for example, Fijj-ImageJ or VLC media player software) at https://doi.org/10.5281/zenodo.999523. Several supplementary figures (Word file) are added to the supplementary information.
