# Supplementary material for: Pigment-Dispersing Factor-expressing neurons convey circadian information in the honey bee brain
Source: Open Biol. 2018 Jan 10;8(1):170224. doi: 10.1098/rsob.170224 (PMC5795053; doi:10.1098/rsob.170224)
Supplement: Supplemental figures and discussion [file rsob170224supp1.docx]

**Supplementary figures**


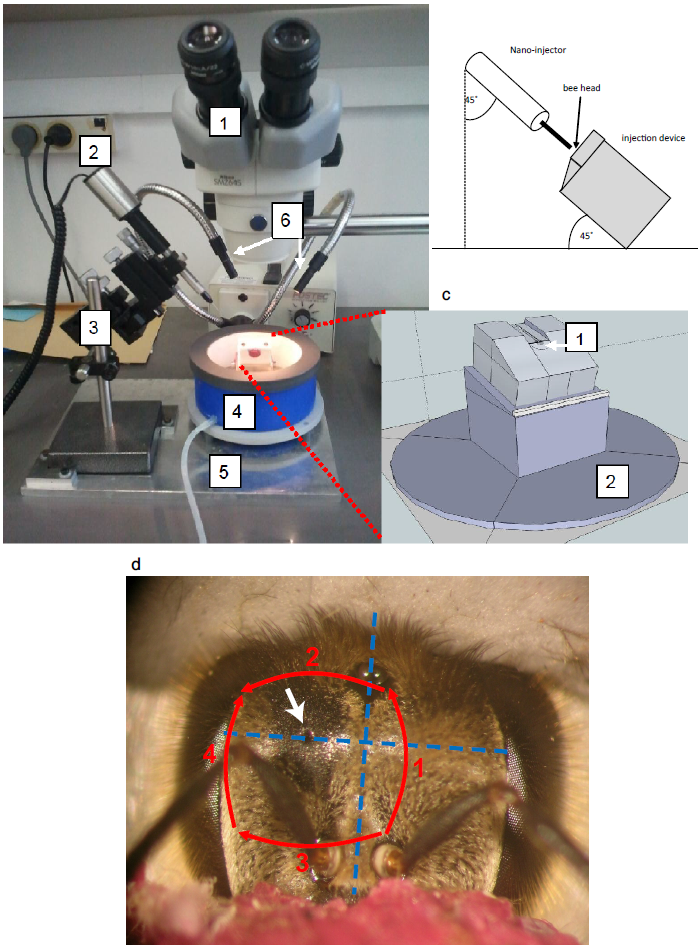


*(a)*

*(b)*

*(c)*

*(d)*

**Figure S1. Setup and procedure for injecting PDF peptide into the honey bee brain without opening a window in the head capsule cuticle.** *(a)* The injection setup: 1 - pole-seated stereo-microscope; 2 - microinjector; 3 - 3D micro-manipulator; 4 - insulated ice container; 5 – an aluminum base; 6 – dual gooseneck fiber optic light source. *(b)* A schematic diagram showing the orientation of the injector and fixation mold during the injection procedure. *(c)* Bee fixation mold. 1 – a slot for the bee neck; 2 – a wide aluminum base allowing efficient chilling of the entire fixation mold. *(d)* Locating the injection site. The blue dashed perpendicular lines depict the gridded lines on the stereo microscope lens (using X40 magnification). These lines were positioned such that the ocellus was placed exactly at the vertical scale mark 25. The arrow points to the injection site which was positioned at scale mark 25 on the left arm of the horizontal grid line. The red, curved lines depict the location and order of incisions made when exposing the injection area for precise site validation at the end of the experiment.


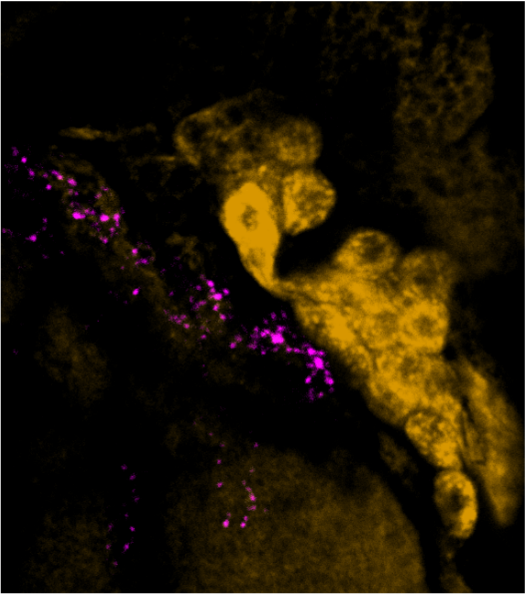

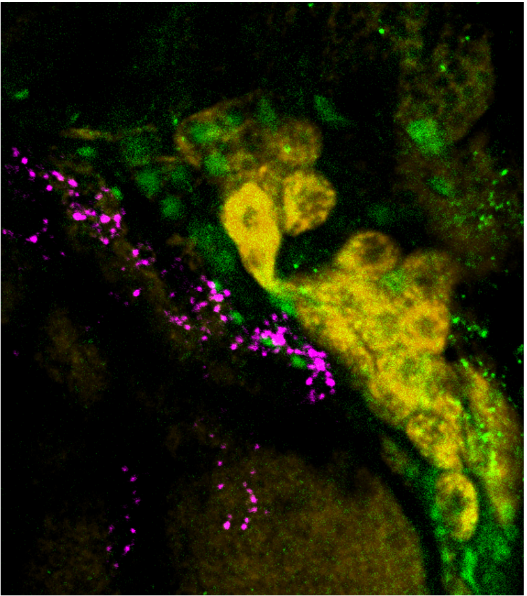

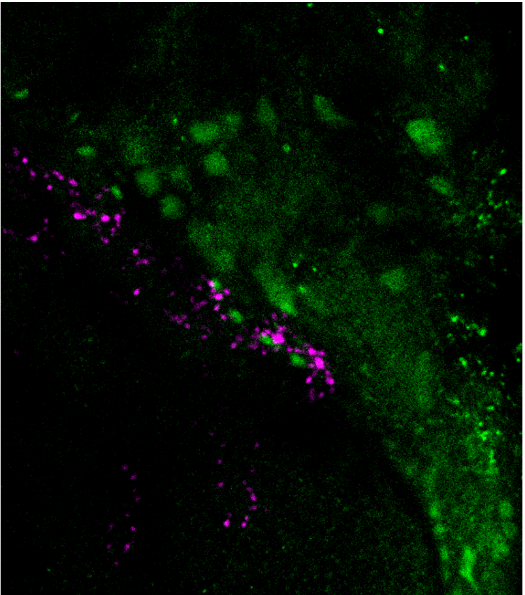


PDF PER

PDF HRP

PDF PER HRP

DN

SNP

SNP

SNP

MCA

MCA

MCA

LCA

LCA

LCA

**Figure S2. PER-positive glia cells accompany the PDF fibres running into the superior neuropil.** Single confocal stack of a vibratome section stained with anti-PER, anti-PDF and anti-HRP. PER-positive glia cells (arrows) can be distinguished from the PER-positive neurons by the lack of HRP. They are located in a row at the border of the superior neuropils (SNP) in close vicinity to the PDF fibres. The PER-positive dorsal neurons (DN) are located dorsally from the PDF fibres and are occasionally contacted by PDF fibres crossing between the medial and lateral calyces (MCA and LCA) to the posterior side of the brain (not visible in this stack). Scale bar 30µm.

**Figure S3. PER-positive glia cells accompany the PDF fibres around the Central and Lateral complex and the oesophageal foramen.** The vibratome sections are triple-labelled with anti-PDF, anti-HRP and anti-PER (left panels: HRP and PDF; right panels: PER and PDF). Upper row: Overlay of three confocal stacks at the level of the central complex, depicting the upper and lower central body (CBU and CBL, respectively) and the lateral and medial bulbs (LBU and MBU) from the lateral complex as well as the median accessory lobe (MAL). PDF fibres are mainly found in the MAL, whereas PER-positive glia cells are located in the areas between the neuropils. Dorsally of the MAL they appear to be touched by PDF fibres that project dorsally. Lower row: Overlay of three confocal stacks at the same level as shown above, but more ventrally. PDF fibres seem to leave the MAL and to follow the dorsal esophageal foramen (ES), which is lined by PER-positive glia cells. HRP labels the neuropils and the somata of neurons. Scale bars: 50 µm.

ES


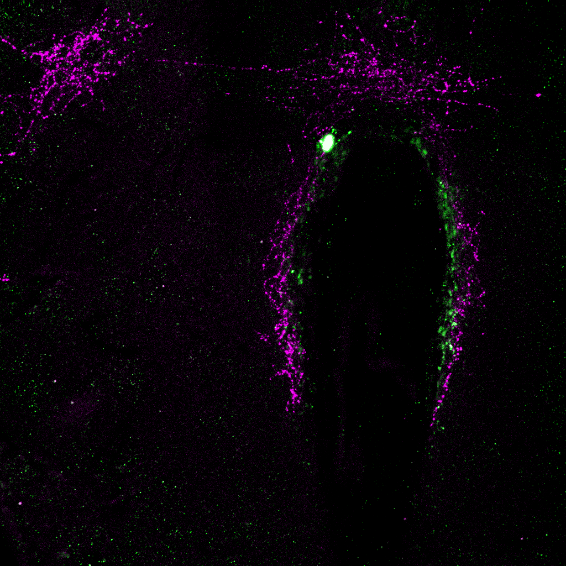

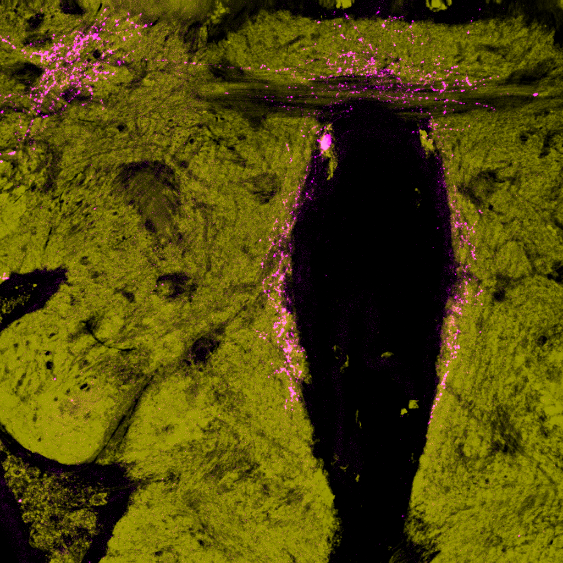

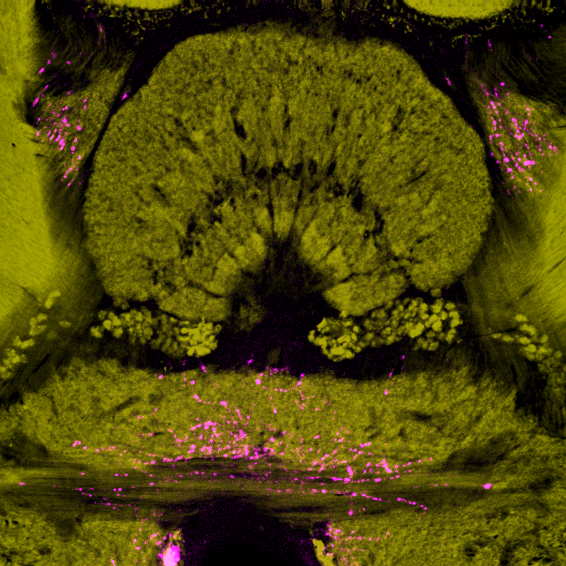

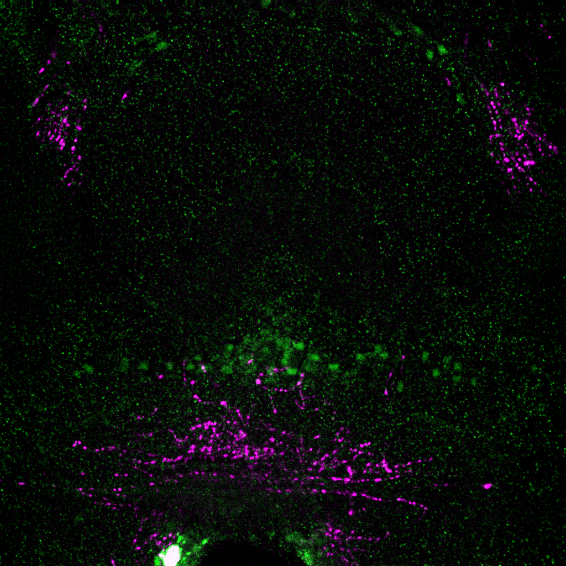


CBU

CBL

CBU

CBL

MBU

LBU

MAL

MAL

ES

HRP PDF

HRP PDF

PDF PER

PDF PER

MAL


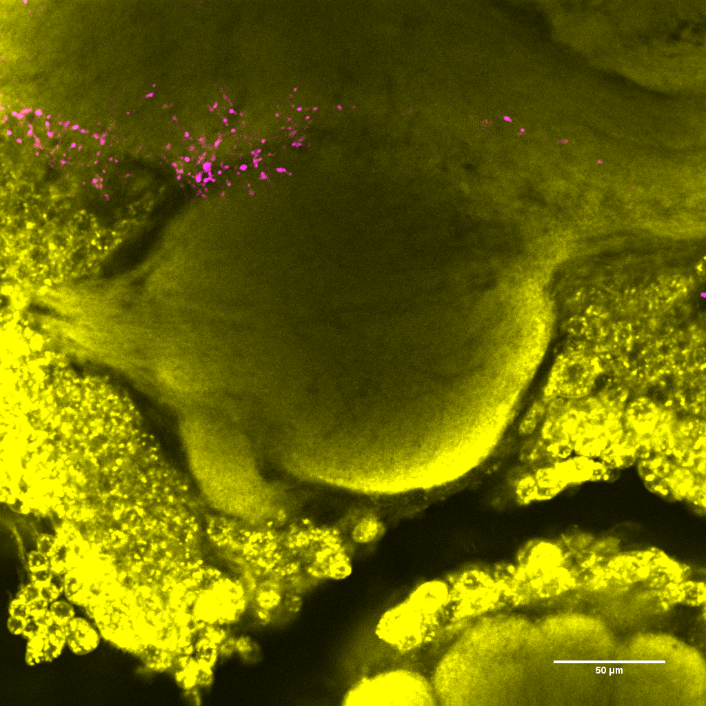


AOTU

HRP PDF

v

l

**Figure S4. PDF fibres close to the anterior optic tubercles.** Single confocal stack of a whole-mount brain stained with anti-PDF (magenta) and anti-HRP (yellow). The PDF fibres that reach into the rim of the anterior optic tubercle (AOTU), which is part of the anterior polarized light input pathway are marked by an arrow. Scale bar: 50 µm. Orientation arrows: l: lateral, v: ventral.

**Figure S5. PDF fibres form “rings” around the vertical lobe and peduncle.** Upper row: Overlay of three confocal stacks at the level of the vertical lobe (VL). PDF fibres form a ring-like network around the VL (magenta arrows). Lower row: Overlay of three confocal stacks at the level of the peduncle (PED). PDF fibres form an inner and outer ring around the PED (magenta arrows). CBU, upper unit of the central body; MCA, medial calyx; LCA, lateral calyx; LVT, lobula valley tract; POC, posterior optic commissure. Horseradish peroxidase (HRP) labels the neuropils and the somata of neurons, DAPI labels the nuclei of glia cells and neurons. Note that around the CBU, VL and PED no neurons are found, but only the nuclei of glia cells.


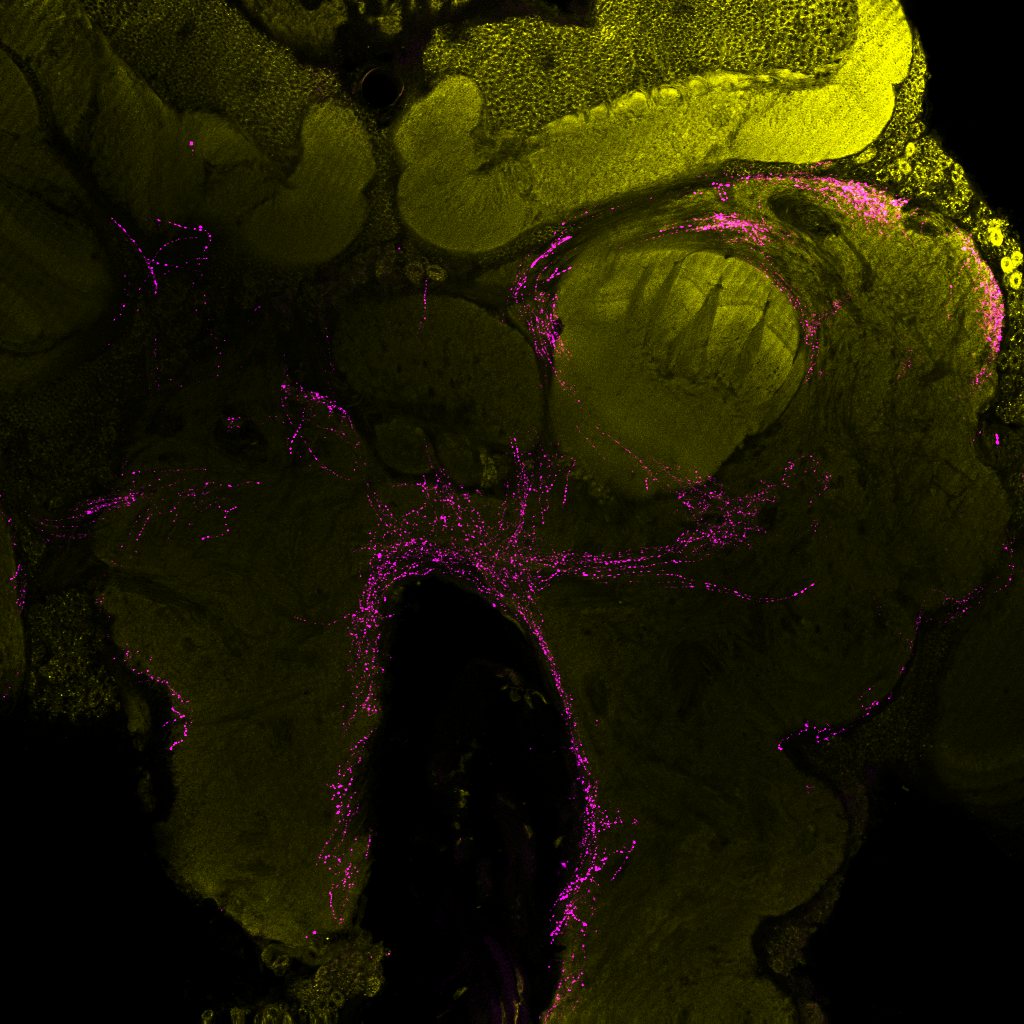

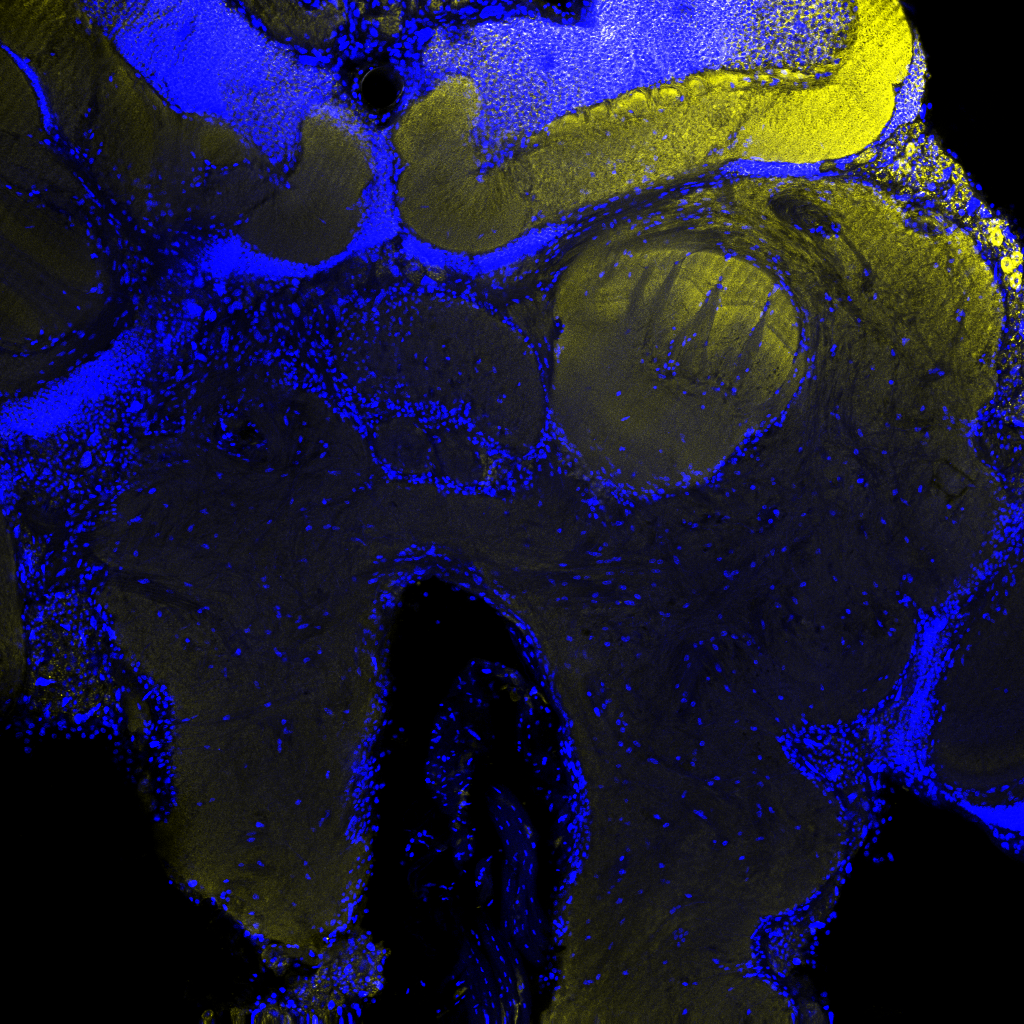

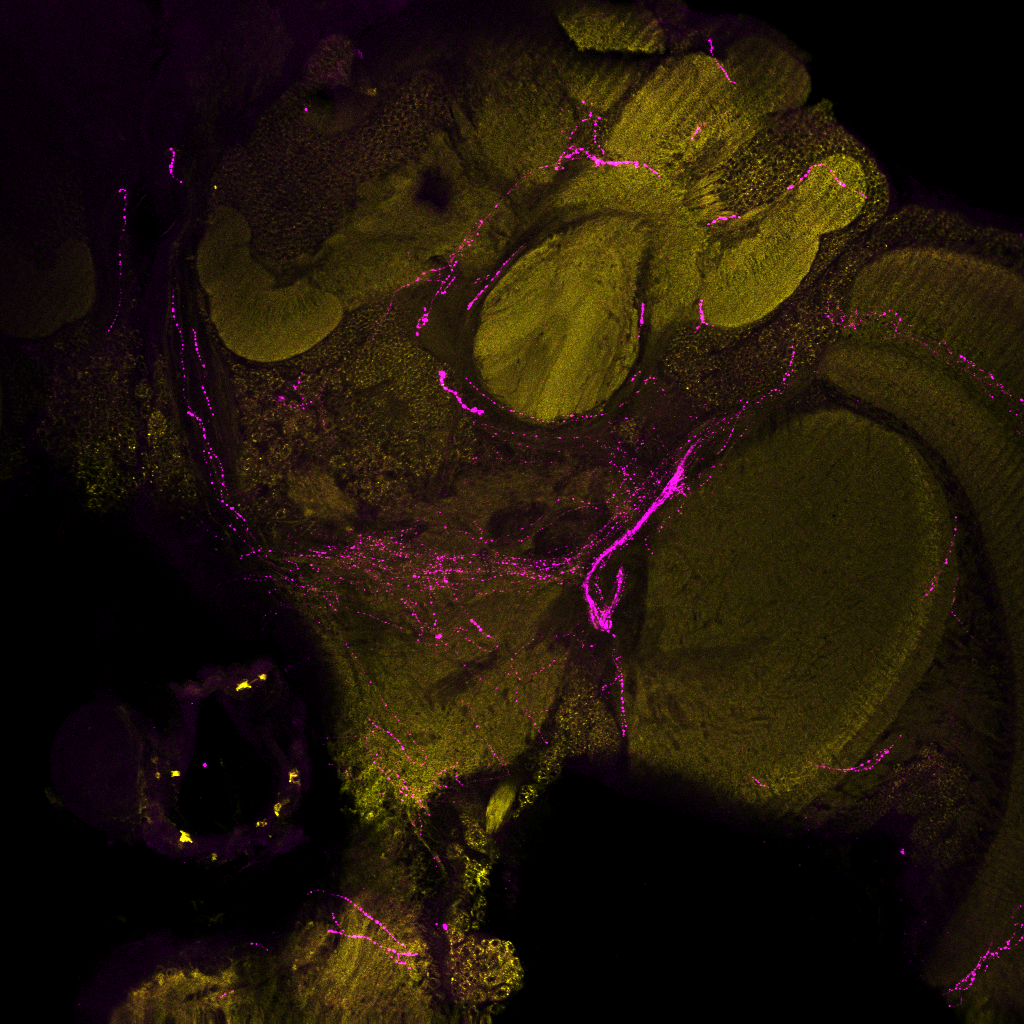

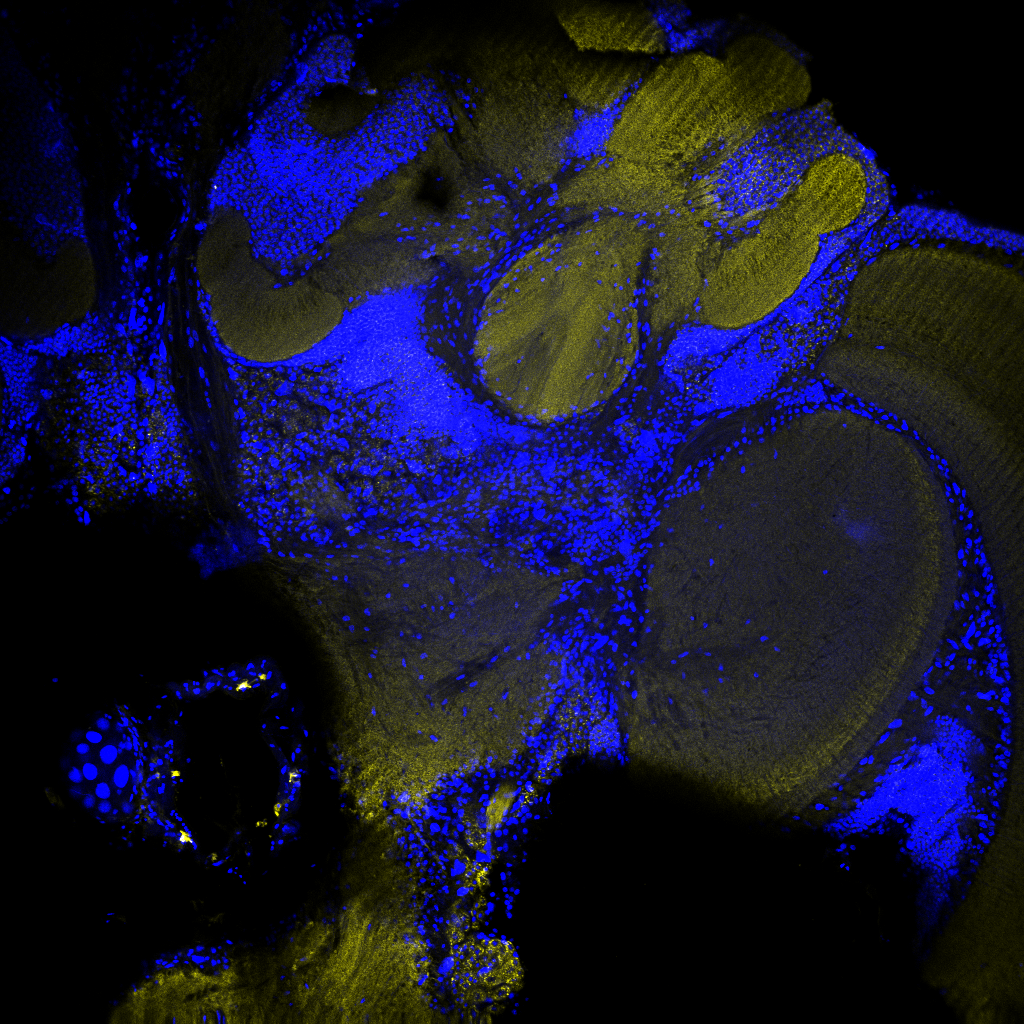


HRP PDF

HRP PDF

HRP DAPI

HRP DAPI

PED

LCA

PED

LCA

MCA

MCA

MCA

MCA

VL

VL

POC

LVT

CBU

CBU

**Figure S6. PDF fibres invading the ocelli.** (*a-b*) Reconstruction of the fibres innervating median and lateral ocelli (MOC and LOC) from a frontal (*a*) and lateral view (*b*). The ones that stem from the median accessory lobe are shown in yellow and the ones that stem from the posterior optic commissure are shown in red. The yellow fibres pass the protocerebral bridge (PB) anteriorly, the red fibres pass it posteriorly. It is unclear, whether they intermingle dorsally of the posterior bridge (arrow). In case they do not intermingle, the fibres stemming from the POC invade the LOC and that stemming from the median accessory lobe invade the MOC. Scale bar: 100 µm. (*c-d*) Confocal stacks of vibratome sections (150 µm and 60 µm) stained with anti-PDF and anti-HRP. (*c*) Several PDF fibers originating from the POC run dorsally towards the ocelli (white arrows). (*d*) Another fiber bundle leaves the median accessory lobe ventrally of the central body (CB), runs between the NO (noduli of the central complex) and continues anterior of the PB (protocerebral bridge) and posterior of the CB towards the medial ocellus (white arrow). Only the edges of the CB are displayed in the picture and we added dotted lines to better outline the neuropils. MCA: medial calyx of the mushroom bodies. Scale bars: 50 µm. Orientation arrows: m: medial, a: anterior, d: dorsal, l: lateral, v: ventral.


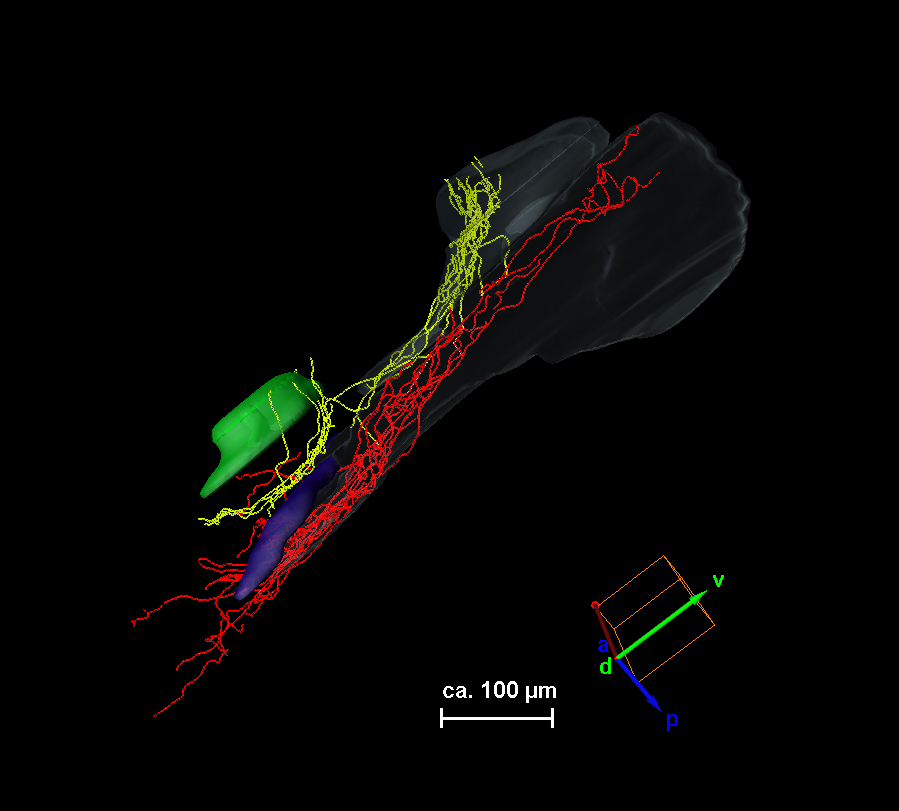


CBU

PB

LOC

MOC

*(b)*


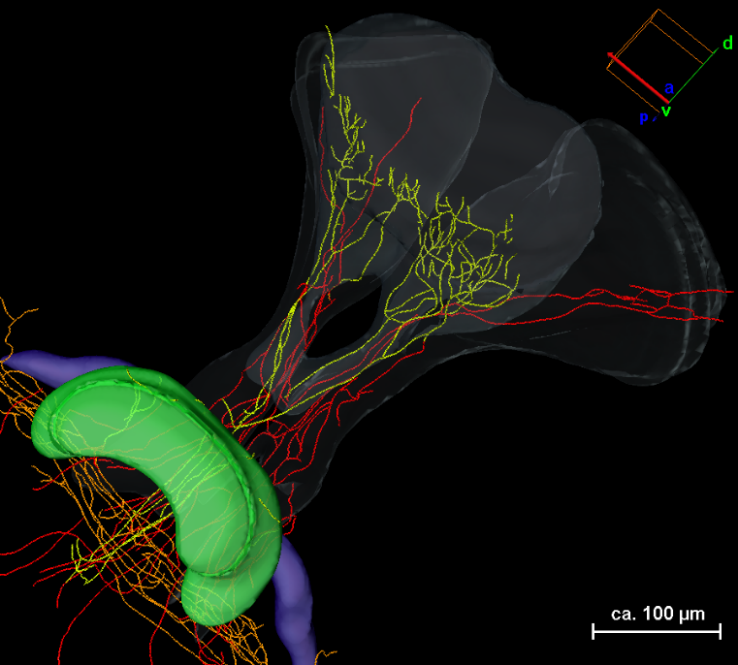


PB

CBU

MOC

LOC

*(a)*


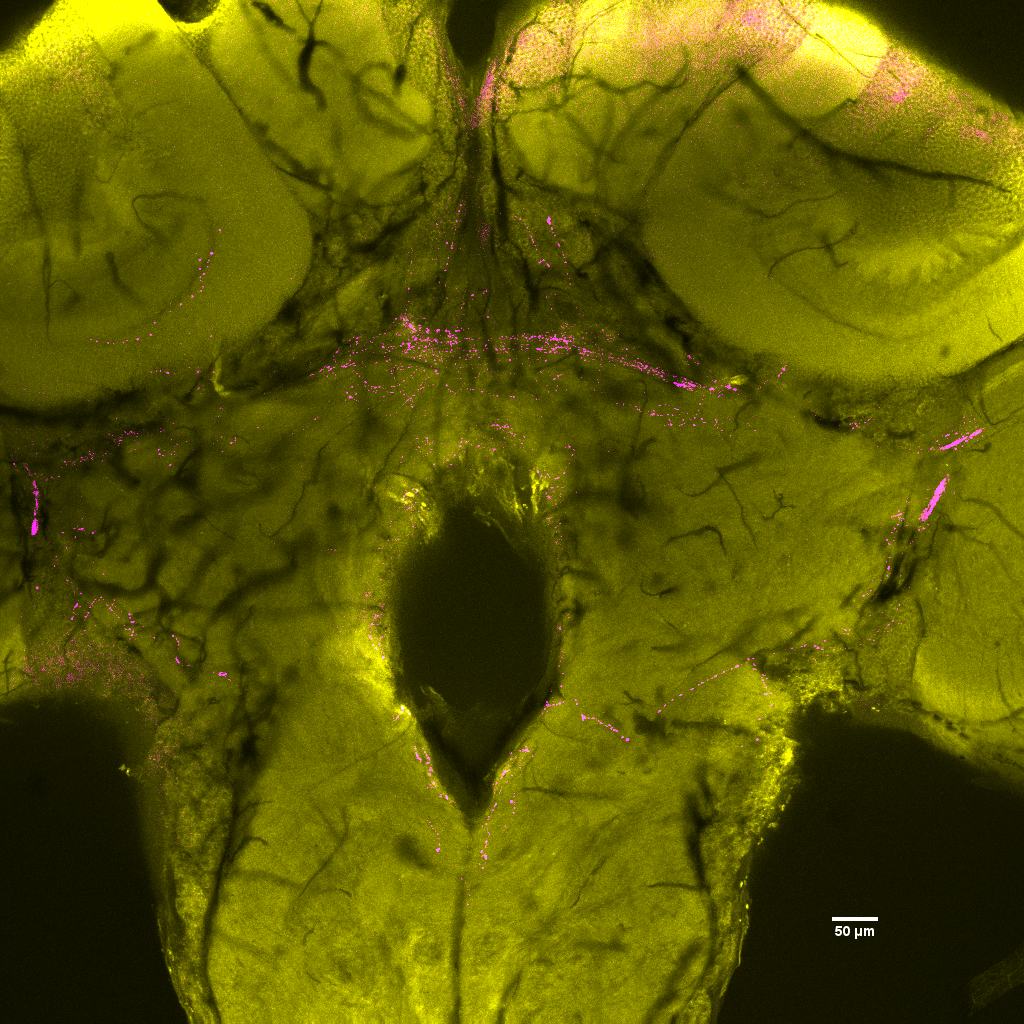


MCA

MCA

v

m

POC

*(c)*


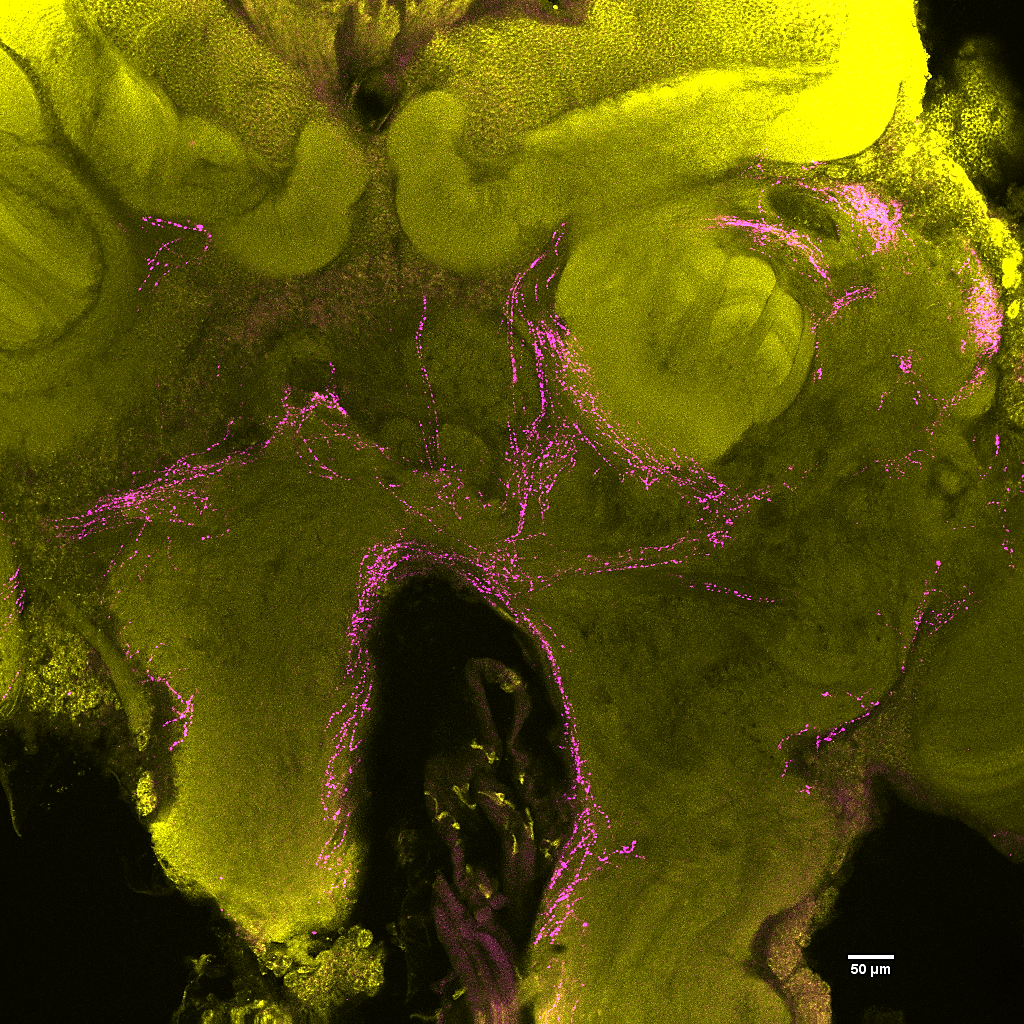


MCA

NO

NO

d

m

a

(CB)

*(d)*


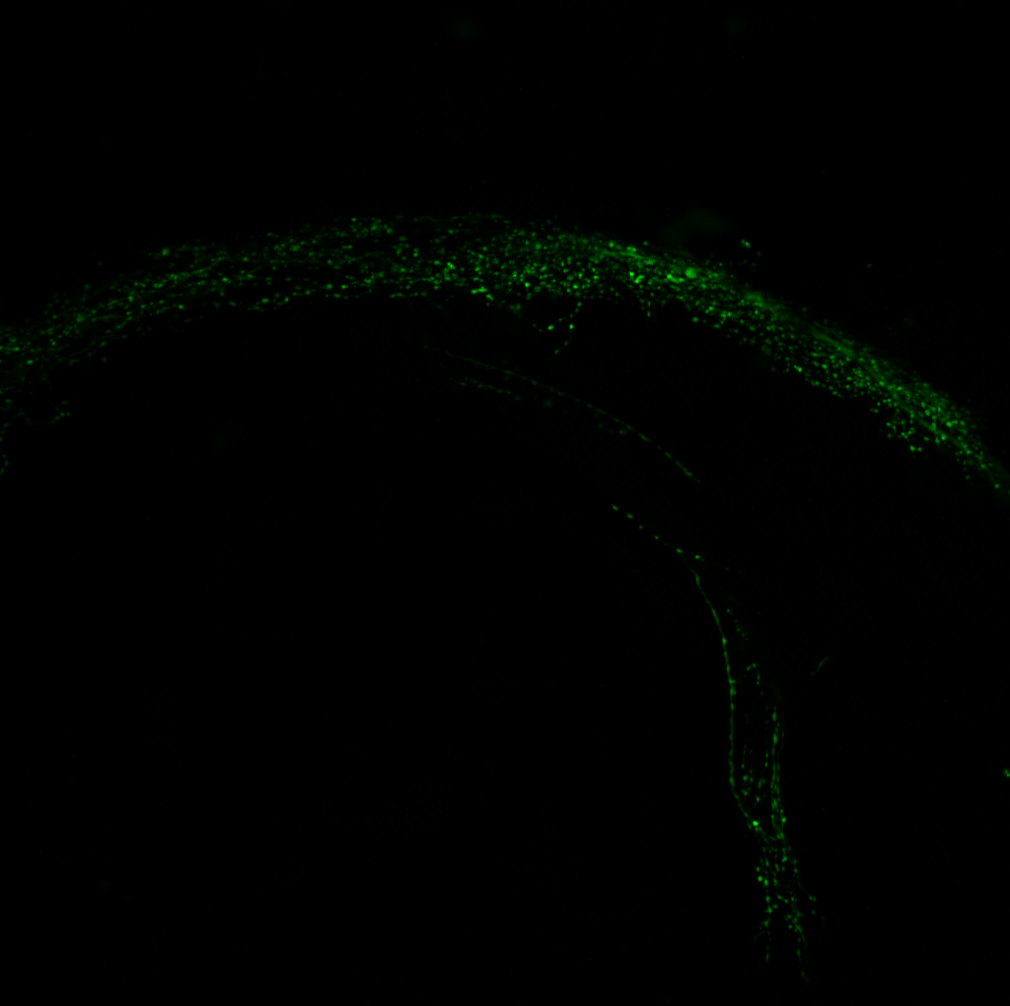

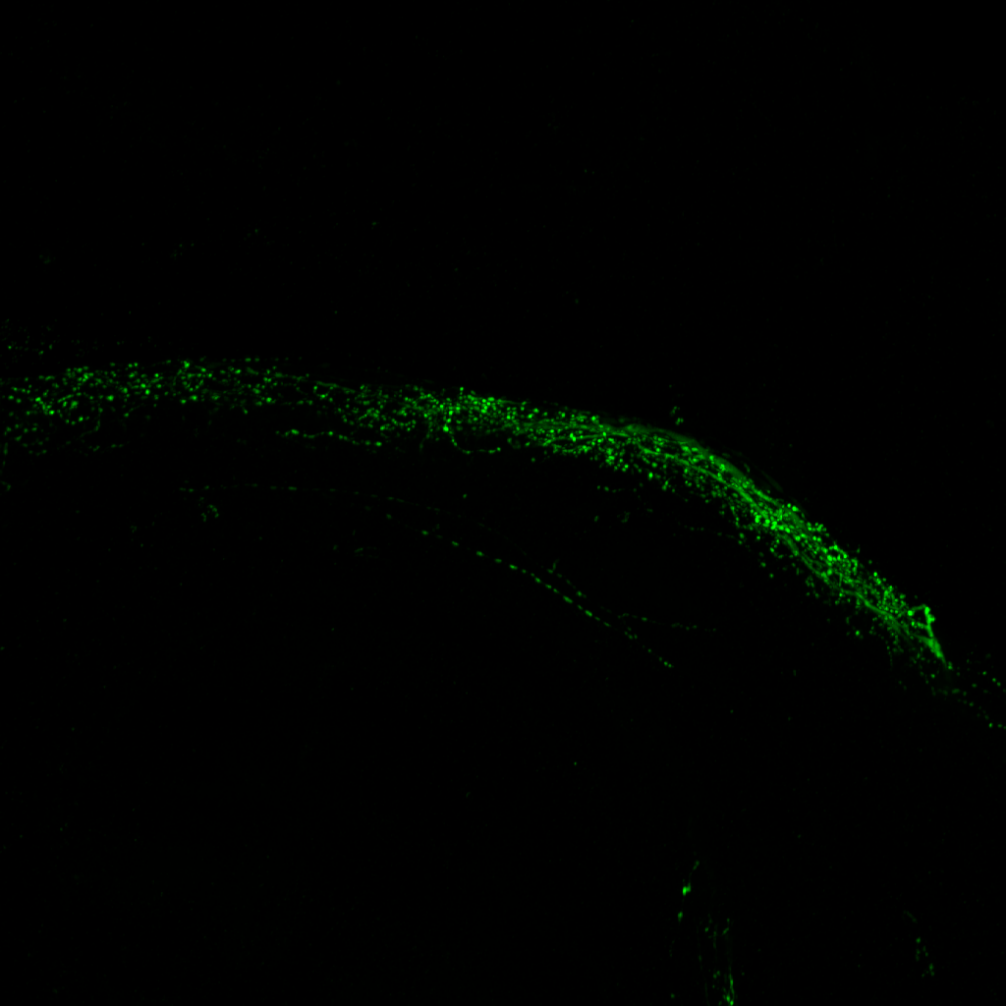


ZT 2

ZT 14

**Figure S7. Example of PDF staining in the fibres below the calyces (area 2 in Fig. 8 and 9) at Zeitgeber Time 2 (ZT2) and Zeitgeber Time 14 (ZT14).** Both pictures represent an overlay of 10 confocal stacks (2µm thick). Confocal settings are identical and no adjustment of brightness or contrast are performed. PDF fibres are very dense at the two time points without any evident differences in neuron structure. Nevertheless, staining intensity is higher at ZT14 than at ZT2.


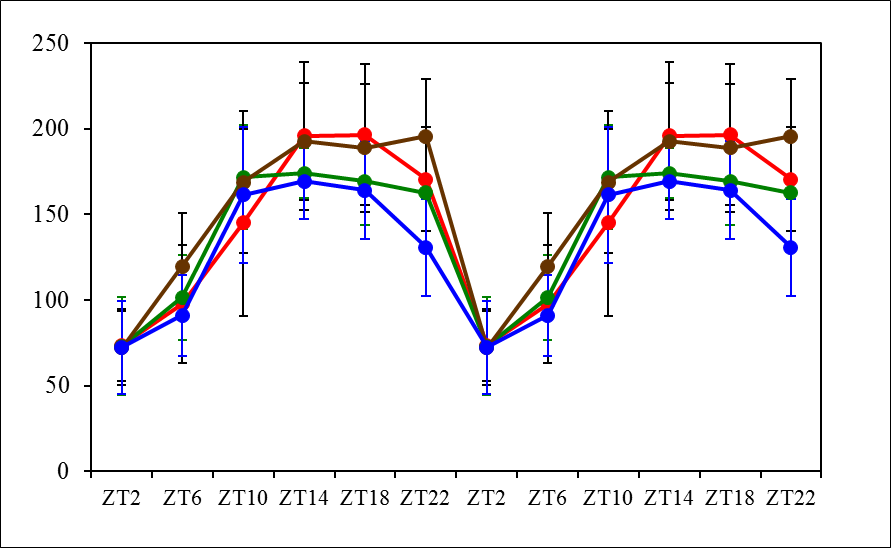


n = 4

Staining intensity (pixel)

nurses

foragers


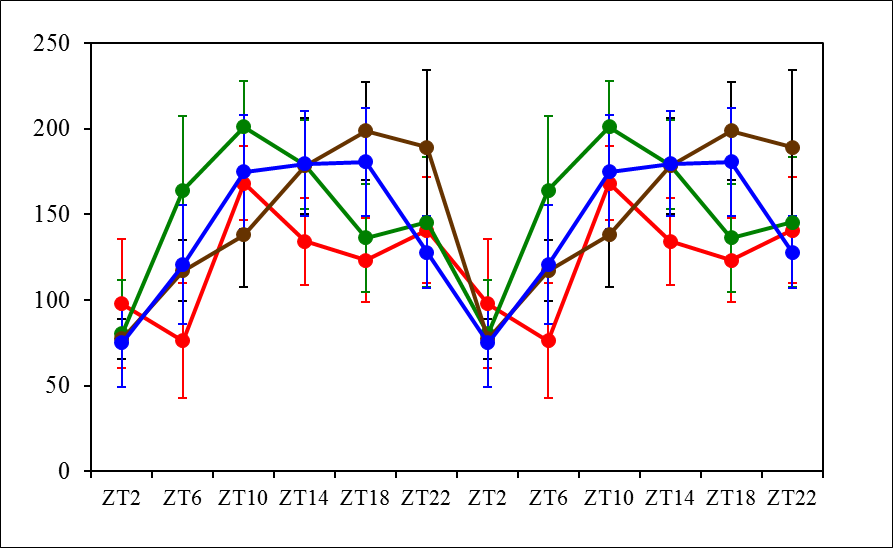


n = 4

p = 0.201

p = 0.330

p = 0.037

p = 0.130

below calyces

median bundle

loop

medulla

p = 0.048

p = 0.136

p = 0.083

p = 0.097

Zeitgeber Time (ZT)

12

0

8

4

16

20

12

0

8

4

16

20

**Figure S8. PDF oscillations under light-dark conditions in nurses and foragers of the second experiments with bees from Jerusalem (6 time points per day).** The bars on top show the LD schedule, with white bars indicating light and black bars indicating darkness. For clarity the entire cycle is repeated. A highly synchronous cycling of PDF can be seen in all structures of nurses, whereas the cycling appeared less synchronous in foragers. ANOVA revealed a significant influence of time of day on PDF-immunostaining intensity in the fibres ventrally of the calyces in nurses and in the loop in foragers (p values in the diagrams; colours correspond to the colours of the curves). As indicated in the left top corners of the diagrams we could evaluate for most time points 4 brains (only one hemisphere per brain). However, at ZT10 for nurses and ZT22 for foragers we had only 3 hemispheres (from 3 brains) that could be evaluated.
